# Proposal for targeted, neo-evolutionary-oriented secondary prevention of early-onset endometriosis and adenomyosis. Part II: medical interventions

**DOI:** 10.1093/humrep/dead206

**Published:** 2023-11-10

**Authors:** Paolo Vercellini, Veronica Bandini, Paola Viganò, Deborah Ambruoso, Giulia Emily Cetera, Edgardo Somigliana

**Affiliations:** Department of Clinical Sciences and Community Health, Academic Centre for Research on Adenomyosis and Endometriosis, Università degli Studi, Milano, Italy; Gynecology Unit, Fondazione IRCCS Ca’ Granda Ospedale Maggiore Policlinico, Milano, Italy; Department of Clinical Sciences and Community Health, Academic Centre for Research on Adenomyosis and Endometriosis, Università degli Studi, Milano, Italy; Department of Clinical Sciences and Community Health, Academic Centre for Research on Adenomyosis and Endometriosis, Università degli Studi, Milano, Italy; Gynecology Unit, Fondazione IRCCS Ca’ Granda Ospedale Maggiore Policlinico, Milano, Italy; Department of Clinical Sciences and Community Health, Academic Centre for Research on Adenomyosis and Endometriosis, Università degli Studi, Milano, Italy; Department of Clinical Sciences and Community Health, Academic Centre for Research on Adenomyosis and Endometriosis, Università degli Studi, Milano, Italy; Gynecology Unit, Fondazione IRCCS Ca’ Granda Ospedale Maggiore Policlinico, Milano, Italy; Department of Clinical Sciences and Community Health, Academic Centre for Research on Adenomyosis and Endometriosis, Università degli Studi, Milano, Italy; Gynecology Unit, Fondazione IRCCS Ca’ Granda Ospedale Maggiore Policlinico, Milano, Italy

**Keywords:** endometriosis, adenomyosis, chronic pelvic pain, menstruation, ovulation, oral contraceptives, progestogens, laparoscopy, central sensitization

## Abstract

According to consistent epidemiological data, the slope of the incidence curve of endometriosis rises rapidly and sharply around the age of 25years. The delay in diagnosis is generally reported to be between 5 and 8years in adult women, but it appears to be over 10 years in adolescents. If this is true, the actual onset of endometriosis in many young women would be chronologically placed in the early postmenarchal years. Ovulation and menstruation are inflammatory events that, when occurring repeatedly for years, may theoretically favour the early development of endometriosis and adenomyosis. Moreover, repeated acute dysmenorrhoea episodes after menarche may not only be an indicator of ensuing endometriosis or adenomyosis, but may also promote the transition from acute to chronic pelvic pain through central sensitization mechanisms, as well as the onset of chronic overlapping pain conditions. Therefore, secondary prevention aimed at reducing suffering, limiting lesion progression, and preserving future reproductive potential should be focused on the age group that could benefit most from the intervention, i.e. severely symptomatic adolescents. Early-onset endometriosis and adenomyosis should be promptly suspected even when physical and ultrasound findings are negative, and long-term ovulatory suppression may be established until conception seeking. As nowadays this could mean using hormonal therapies for several years, drug safety evaluation is crucial. In adolescents without recognized major contraindications to oestrogens, the use of very low-dose combined oral contraceptives is associated with a marginal increase in the individual absolute risk of thromboembolic events. Oral contraceptives containing oestradiol instead of ethinyl oestradiol may further limit such risk. Oral, subcutaneous, and intramuscular progestogens do not increase the thromboembolic risk, but may interfere with attainment of peak bone mass in young women. Levonorgestrel-releasing intra-uterine devices may be a safe alternative for adolescents, as amenorrhoea is frequently induced without suppression of the ovarian activity. With regard to oncological risk, the net effect of long-term oestrogen–progestogen combinations use is a small reduction in overall cancer risk. Whether surgery should be considered the first-line approach in young women with chronic pelvic pain symptoms seems questionable. Especially when large endometriomas or infiltrating lesions are not detected at pelvic imaging, laparoscopy should be reserved to adolescents who refuse hormonal treatments or in whom first-line medications are not effective, not tolerated, or contraindicated. Diagnostic and therapeutic algorithms, including self-reported outcome measures, for young individuals with a clinical suspicion of early-onset endometriosis or adenomyosis are proposed.

## Introduction

Based on accumulating and consistent epidemiological data, the slope of the incidence curve of endometriosis rises rapidly and sharply around the age of 25 years (Parazzini *et al.*, 2020). However, while the delay in diagnosis is generally reported to be between 5 and 8 years in adult women (Chapron *et al.*, 2019; Horne and Missmer, 2022; Allaire *et al.*, 2023), it appears to be >10 years in young women (Becker *et al.*, 2022; Pino *et al.*, 2023). This is probably due to multiple reasons, including lack of awareness of the condition during adolescence among clinicians; normalization of pain by family, friends and schoolmates; erroneous exclusion of endometriosis after a negative ultrasound (US) scan; hesitancy of gynaecologists to order pelvic MRI; and reluctance of young women to undergo laparoscopy for visual diagnosis (Brosens *et al.*, 2013; Youngster *et al.*, 2013; ACOG, 2018b; Myszko *et al.*, 2020).

Irrespective of the underlying reasons, if the reported data on diagnostic delay are reliable, the actual onset of endometriosis in many young women would occur in the early postmenarchal years (beginning of peak incidence, 25 years, minus diagnostic delay in young women, >10 years, equals endometriosis onset at ∼15 years of age). In fact, data on the incidence of endometriosis are mostly based on surgical diagnosis, so the actual slope should be shifted to the left anyway, as lesions reasonably develop and causes pain symptoms some years before definitive identification (Kvaskoff *et al.*, 2013). Therefore, attention should be focused on severely symptomatic adolescents, who are at increased risk of being affected by the disease and could benefit most from secondary preventive interventions to reduce suffering and limit lesion progression.

In the first part of this opinion piece, we described the remarkable epidemiological changes in reproductive patterns that have occurred over the last two centuries, leading to an extraordinary increase in the number of ovulatory menstrual cycles throughout the reproductive period and, perhaps most importantly, in the early postmenarchal years. Regardless of any additional contributing cause, if ovulatory menstruations play a role in the development of endometriosis and adenomyosis, the 10-fold increase in their number between menarche and first full-term pregnancy since pre-industrial times (Eaton *et al.*, 2002) must be carefully considered as a risk factor that can be modified.

In recent decades, several medical interventions have been proposed to control the manifestations of endometriosis and adenomyosis, including short-term hormonal suppression of ovulation and surgical removal of the anatomical consequences of both diseases. However, these treatments are not curative and may have limited efficacy. Therefore, the best therapeutic strategy to achieve the above goals is still under debate.

In the second part of this opinion piece, we (i) propose the concept ‘*suspect endometriosis and adenomyosis in severely symptomatic young women, even when physical and US findings are negative, until proven otherwise*’, with the aim of limiting the diagnostic delay and the associated consequences; (ii) support a secondary prevention strategy by long-term menstrual suppression commencing hormonal therapies promptly after clinical suspicion or imaging evidence of one or both diseases, with the aim of relieving symptoms, avoiding lesion progression, and preserving future reproductive potential.

The literature search strategy and selection criteria for the evidence reviewed in this article are described in part I (Vercellini *et al.*, 2023).

## Retrograde menstruation and the risk of endometriosis: a quantitative or qualitative issue?

The main criticism of the implantation theory is the discrepancy between the almost universal phenomenon of retrograde menstruation during the reproductive years and the relatively low endometriosis prevalence in the general population (Parazzini *et al.*, 2020). This gap has been interpreted both quantitatively, i.e. endometriosis only develops when the amount of refluxed erythrocytes and endometrial fragments exceeds the scavenging capacity of the peritoneal macrophages and iron-transport proteins (D'Hooghe and Debrock, 2002; Wyatt *et al.*, 2023), and qualitatively, that is, endometriosis only develops when pathological endometrium, carrying specific abnormalities that confer to the shedding glands an increased capacity to implant on the peritoneum, to proliferate, and to infiltrate tissues, reaches the pelvis (Vinatier *et al.*, 2000).

The ‘quantitative’ hypothesis is supported by a large amount of data demonstrating dysregulated iron homeostasis in endometriosis patients, caused by (i) an excess of erythrocytes entering the pelvis during menses exceeding the degradation capacity of pelvic macrophages (Donnez *et al.*, 2016), and/or (ii) aberrant expression of iron-transport proteins, i.e. a defective protective iron-sequestration mechanism (Wyatt *et al.*, 2023). The iron overload resulting from repeated bleeding episodes would trigger local oxidative stress, maintain a pro-inflammatory state, induce anomalous resistance to ferroptosis (i.e. a form of iron-dependent, non-apoptotic programmed cell death, caused by toxic lipide peroxidation-mediated membrane damage) and progesterone resistance, and favour ectopic endometrium proliferation (Ng *et al.*, 2020a; Li *et al.*, 2023; Ma *et al.*, 2023; Wyatt *et al.*, 2023).

The ‘qualitative’ hypothesis is based on the differential expression of many molecules, at the gene and/or protein level, observed in the eutopic endometrium of patients with endometriosis compared with that of individuals without the disease (Vinatier *et al.*, 2000; Ulukus *et al.*, 2006). Endometrial abnormalities have been identified in several cellular processes, including, but not limited to, proteolysis, angiogenesis, oestrogen synthesis, response to progesterone, and apoptosis (Viganò *et al.*, 2023). In addition, eutopic and ectopic endometrial oligoclones carrying somatic mutations in cancer driver genes have been consistently detected in the epithelial cells of the mucosa of individuals with endometriosis and adenomyosis.

Anglesio *et al.* (2017) studied non-ovarian, infiltrating endometriotic lesions from 39 patients and found somatic mutations in the majority of them, including cancer driver mutations in *ARID1A*, *PIK3CA*, *KRAS*, or *PPP2R1A* in five cases (Anglesio *et al.*, 2017). After sequencing epithelial cells from 107 endometriomas as well as 82 samples of normal endometrium from control subjects with benign gynaecological conditions, Suda *et al.* (2018) suggested that ovarian endometriosis also develops from clonal expansion of endometrial epithelial cells carrying distinct somatic mutations within cancer-associated genes, in particular *KRAS*.

Orr *et al.* (2023a) have recently reported that the presence of *KRAS* mutations in endometriotic lesions excised in a series of 122 patients, was associated with greater anatomic disease severity and increased surgical difficulty. Somatic *KRAS* mutations were more frequently detected in subjects with infiltrating fibrotic lesion or endometrioma only (11/19; 58%) and mixed subtypes (40/66; 61%), than in those with superficial implants only (13/37; 35%).

Instead of exploring the presence of somatic cancer-driver mutations in the same endometriotic lesion type in different patients, Praetorius *et al.* (2022) analysed mutations across different lesions types excised from the same patient. Alterations on cancer-associated genes were detected in lesions from 13 of 27 study subjects, with more lesions again affected by *KRAS* changes (15/53 lesions in 6 cases). In nine of these 13 patients mutations were identical across distinct lesions. These findings are consistent with individual lesions being oligoclonal, with different lesions within the same patient sharing a common cell lineage, and with a metastatic model of disease propagation (Praetorius *et al.*, 2022).

Recurring *KRAS* mutations were found by Inoue *et al.* (2019) also in 37.1% (26/70) of patients with adenomyosis. Oligoclonality was demonstrated, with some mutations identified in co-occurring endometriosis.

Although somatic mutations in cancer-associated genes are per se insufficient for malignant derailment, several lines of evidence support the hypothesis that intrauterine, deep-invaginating endometrial crypts harbouring these mutations may be selectively advantaged (Bulun, 2022). In particular, the emergence of distinct *KRAS*-mutated clonal epithelial cell populations characterized by epigenetically downregulated progesterone receptors, enhanced survival and proliferative capacity, and invasiveness, may be considered a key component of the molecular pathogenesis of both adenomyosis and endometriosis (Anglesio *et al.*, 2017; Suda *et al.*, 2018; Inoue *et al.*, 2019; Praetorius *et al.*, 2022). Identical *KRAS* mutations have been detected in epithelial cells from the basalis layer of eutopic endometrium, adjacent adenomyosis foci and coexistent endometriotic lesions, supporting a common pathogenic process for both adenomyosis and endometriosis (Bulun *et al.*, 2021).

However, both positions have been questioned. On the one hand, the ‘universality’ of retrograde menstruation has not been definitively demonstrated, if it must entail the perimenstrual presence of endometrial fragments in addition to blood in the peritoneal fluid (see Part I of this article – Vercellini *et al.*, 2023). On the other hand, the various abnormalities found in the endometrium of patients with endometriosis have been considered as a potential epiphenomenon of the disease itself (Guo *et al.*, 2023; Viganò *et al.*, 2023).

Despite the above criticisms, a now vast body of evidence supports the notion that repetitious episodes of ovulatory menses, by favouring both excessive bleeding and intramyometrial entrapment of mutated cell populations as well as their extra-uterine dissemination via transtubal retrograde flow, may constitute the early steps in the establishment and persistence of most adenomyosis and endometriosis cases (Donnez *et al.*, 2016; Ng *et al.*, 2020a; Bulun *et al.*, 2021, 2023; Bulun, 2022; Kobayashi, 2023; Wyatt *et al.*, 2023). Importantly, *KRAS* mutations have been suggested to confer resistance to ferroptosis in lung cancer (Bartolacci *et al.*, 2022) and pancreatic ductal adenocarcinoma (Li *et al.*, 2022). The relationship between *KRAS* mutations and resistance to ferroptosis should also be investigated in endometriosis and adenomyosis. A sequence of events starting with iron overload that could lead to *KRAS* mutations and hence resistance to ferroptosis, would combine the quantitative and qualitative theories. In addition, this would further emphasize the importance of pharmacologically reducing the exposure of the uterine wall and the pelvis to excessive amounts of blood as a potential source of free iron to reduce the risk of the emergence of oxidative stress-generated mutated endometrial oligoclones.

Therefore, whether the development of endometriosis from retrograde menstruation is a quantitative or a qualitative problem or a synergistic effect between the two, transtubal reflux of sloughed endometrium should be limited as much as possible in patients with even a suspicion of the disease. Of relevance here, the endometrium can reach a thickness of 12–16 mm during the late secretory phase (Nalaboff *et al.*, 2001; D’Arpe *et al.*, 2016), whereas the average endometrial thickness is generally 3–5 mm during protracted use of combined oral contraceptives (COCs) (ESHRE Capri Workshop Group, 2001; D’Arpe *et al.*, 2016) and even lower during progestogen monotherapy (ESHRE Capri Workshop Group, 2001; Laganà *et al.*, 2017). Given the state of endometrial atrophy achieved with prolonged use of COCs or progestogens, it is tempting to speculate that the menstrual effluent associated with bleeding when using these drugs may not predispose to the development of endometriosis to the same extent as that associated with physiological menses. Indeed, the remarkable reduction in the amount of menstrual flow in COC users (Mansour *et al.*, 2017) and the generally scanty irregular bleeding in progestogen users (Vercellini *et al.*, 2016a,b) should also result in a proportional reduction in the amount of transtubal retrograde bleeding.

## Suppression of repetitive ovulatory menstruation in symptomatic adolescents: remediating the adaptation-evolution mismatch as secondary prevention of early-onset endometriosis and adenomyosis

The current reproductive pattern is the result of a profound and inalienable social evolution in favour of women’s professional advancement, economic and psychological independence, self-determination of their personal future, and an increasingly participatory and decision-making role in all aspects of life. There is no going back.

To counteract the rising incidence of diseases associated with decades of uninterrupted ovulatory menstruation and excessive oestrogen exposure (i.e. ovarian, endometrial and breast cancer and endometriosis), Eaton *et al.* (2002) proposed early endocrinological interventions to reduce average serum oestrogen levels and simulate the ancestral hormonal milieu by inducing pseudopregnancy with COCs.

However, there is currently no evidence to suggest that pharmacological suppression of ovulatory menstruation in all adolescent women at average risk as a primary preventive measure would significantly reduce the incidence of endometriosis later in life (Vercellini *et al.*, 2011). Furthermore, considering that the prevalence of endometriosis in the general female population of reproductive age is ∼5% (Parazzini *et al.*, 2020), several young women would be treated unnecessarily to potentially prevent or delay a single case of endometriosis. In addition, the financial implications of such an approach would be burdensome for public health systems, with improper opportunity costs, especially given the uncertain benefits. Finally, the acceptability of and adherence to systematic induction of amenorrhoea immediately after menarche even in asymptomatic girls would likely be very limited, undermining the effectiveness of this approach.

### Identifying adolescents at risk of early-onset endometriosis and adenomyosis

A completely different strategy would be to focus on a selected population subgroup of adolescents with strong clinical indicators of early-onset endometriosis and adenomyosis (Table 1). The likelihood of severe menstrual pain increases significantly with lower menarchal age (Hoppenbrouwers *et al.*, 2016), and younger patients have higher levels of dysmenorrhoea, dyspareunia and non-cyclic pelvic pain than older ones (Treloar *et al.*, 2010; DiVasta *et al.*, 2018; Wüest *et al.*, 2023). Along this line, Lund *et al.* (2022) recently observed a robust inverse relationship between age at menarche and chronic pain outcomes in adult women. Each additional year (increase) in age at menarche was associated with a ≥5-year reduction in the risk of future chronic pain symptoms. The higher oestrogen levels found in menstruators with early menarche persist for years after puberty and may promote the development of chronic pain. Thus, early menarche could be seen as a proxy measure of elevated and pro-inflammatory oestrogen exposure during development (Lund *et al.*, 2022). Furthermore, acute dysmenorrhoea episodes early after menarche, if repeated unremittingly, may not only be an indicator of ensuing endometriosis and adenomyosis, but may also promote the transition from acute to chronic pelvic pain through central sensitization mechanisms and the onset of chronic overlapping pain conditions (Jarrell and Arendt-Nielsen, 2016a,b; de Arruda *et al.*, 2022).

**Table 1.** Indicators that should raise the suspicion of early-onset endometriosis in adolescents despite normal physical examination and pelvic ultrasound findings.*

1. History of endometriosis in first-degree relatives
2. Early menarche (<12 years)
3. Regular menstrual cycles soon after menarche
4. Severe or worsening dysmenorrhoea (≥7 on a 0- to 10-point numerical rating scale), especially when associated with nausea, vomiting, and diarrhoea
5. Non-response to standard NSAIDs
6. Abundant menstrual flows
7. Mid-cycle or acyclic pain
8. Irritative gastrointestinal symptoms
9. Genitourinary symptoms
10. Deep dyspareunia
11. Academic absenteeism and presenteeism
12. Reduced performance in sports and extracurricular activities
13. Depression, anxiety, deteriorated psychosocial functioning

* ACOG (2018b), DiVasta *et al.* (2018, 2021), Geysenbergh *et al.* (2017), Martire *et al.* (2020, 2023), Wüest *et al.* (2023), and Zannoni *et al.* (2014).

**Figure 1.**
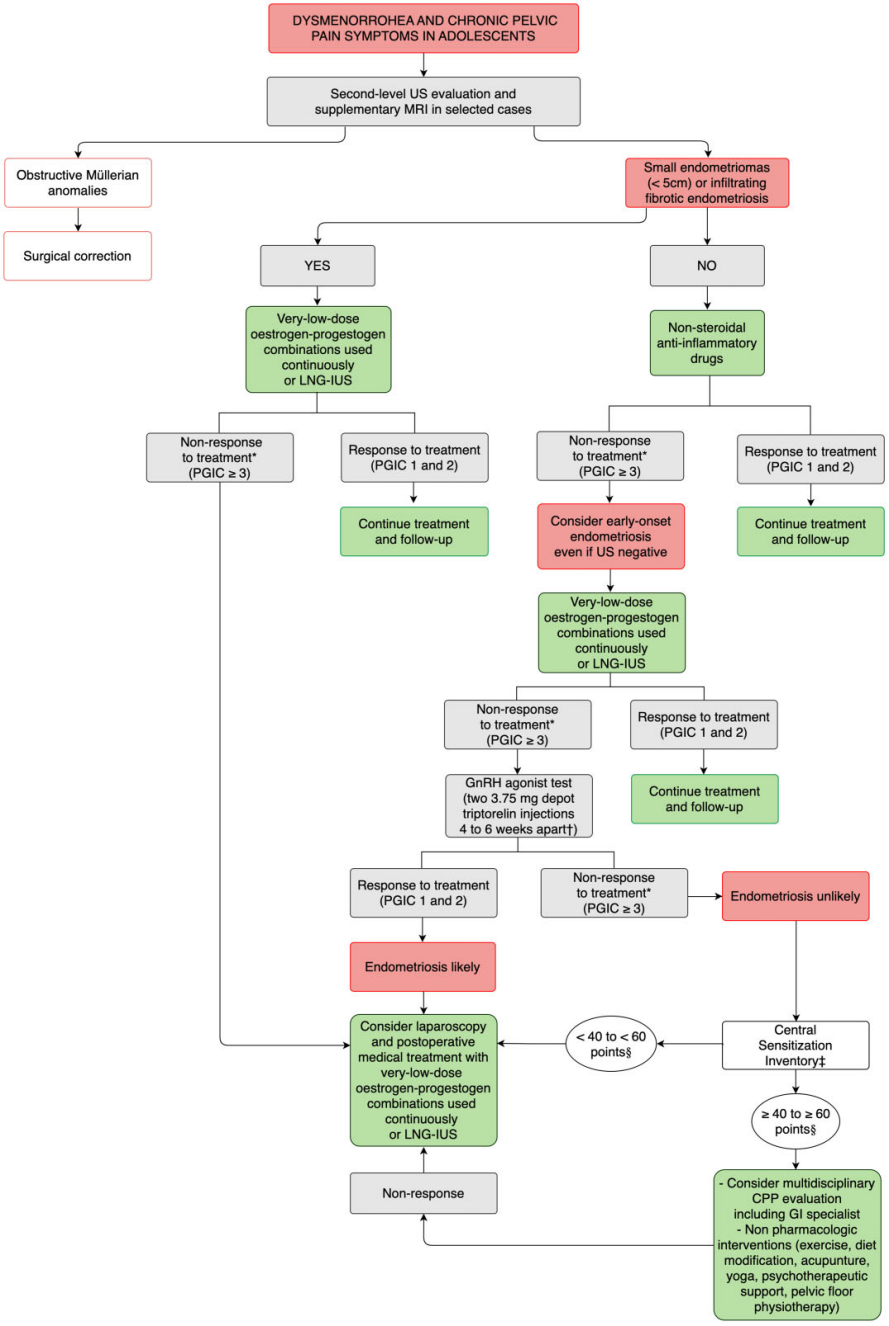
**Proposal for a diagnostic and therapeutic algorithm, including self-reported outcome measures, for the young menstruator with severe dysmenorrhoea, chronic, acyclic pelvic pain symptoms, and a clinical suspicion of early-onset endometriosis who prefers medical suppression of menses to surgery, and accepts, tolerates, and has no contraindications to long-term hormonal treatment.** US, ultrasonographic scan; MRI, magnetic resonance imaging; LNG-IUS, levonorgestrel-releasing intra-uterine system; PGIC, patient global impression of change 7-point scale (Guy, 1976; Dworkin *et al.*, 2005); CPP, chronic pelvic pain. *After at least 3-month treatment. ^**†**^Based on data from Vercellini *et al.* (2023a). ^**‡**^Central Sensitization Inventory 0–100 score (Mayer *et al.*, 2012; Orr *et al.*, 2022, 2023b; Cetera *et al.*, 2023a). ^§^Based on data from Neblett *et al.* (2017) and Orr *et al.* (2020, 2023b).

The notion of subclassifying early dysmenorrhoea into primary (painful menses in the absence of pelvic pathology; ACOG, 2018b) and secondary (painful menses due to pelvic pathology or a recognized medical condition; ACOG, 2018b) may be irrelevant, once the presence of specific obstructive genital anomalies that do not completely impede menstrual outflow (i.e. a rudimentary, cavitated, non-communicating rudimentary horn, or a didelphic uterus with imperforate hemi-vagina) is ruled out (Fig. 1). Indeed, a non-invasive diagnosis of endometriosis is currently proposed as the standard of care (Taylor *et al.*, 2018; Agarwal *et al.*, 2019; Chapron *et al.*, 2019; Becker *et al.*, 2022). Given that neither physical examination nor US and MR imaging can reliably exclude the presence of the most common lesion type detected in postmenarchal years, i.e. endometriotic superficial peritoneal implants (Rasp *et al.*, 2022), it seems unclear on what basis painful menstruation can be defined as ‘primary’.

However, with proper history taking and accurate US imaging criteria application, the prevalence of ovarian and infiltrating fibrotic endometriotic lesions in symptomatic young individuals appears to be higher than previously thought (Martire *et al.*, 2023; Millischer *et al.*, 2023). Moreover, contrary to prior assumptions, mild to moderate adenomyosis is also common in the adolescent population complaining of heavy menstrual bleeding and dysmenorrhoea, and its frequent co-existence with endometriosis (Exacoustos *et al.*, 2022) suggests a common pathogenesis.

In a series of 371 young women with severe dysmenorrhoea and heavy menstrual bleeding, Martire *et al.* (2023) found US evidence of endometriosis in over one-third of them. In addition to posterior infiltrating fibrotic endometriosis, mainly focal thickening of the uterosacral ligament (53%) and small ovarian endometriomas (41%), also adenomyosis, mostly in a mild form, was identified in more than half of the women with endometriotic lesions (67/131, 51%).

Using MRI in another large series of 308 adolescents reporting severe dysmenorrhoea unresponsive to non-steroidal anti-inflammatory drugs (NSAIDs), Millischer *et al.* (2023) confirmed a high prevalence of endometriomas, infiltrating and fibrotic endometriosis, and adenomyosis, and observed a linear increase in frequency over time. In the 18–20 year age group, the majority of young menstruators with severe dysmenorrhoea had MRI evidence of endometriosis and/or adenomyosis (endometriomas, 21.5%; posterior infiltrating fibrotic endometriosis, 89.8%; adenomyosis, 21.5%), further supporting the hypothesis that both conditions progress during the early postmenarchal years.

Eventually, two-thirds of adolescents undergoing laparoscopy for severe dysmenorrhoea and chronic pelvic pain symptoms have endometriosis (ACOG, 2018b; Hirsch *et al.*, 2020). Thus, endometriosis must always be suspected and treated promptly when dysmenorrhoea and chronic acyclic abdominopelvic pain do not respond to NSAIDs, interfere with daily and academic activities, and worsen health-related quality of life (Wüest *et al.*, 2023; Table 1).

### Ovulation suppression or surgery for the secondary prevention of early-onset endometriosis and adenomyosis?

As a secondary prevention measure, ovulatory menstruation could be suppressed in severely symptomatic adolescents from the onset of pelvic pain symptoms or US identification of endometriotic and adenomyotic lesions until conception seeking (ACOG, 2018a). The primary goal of this neo-evolutionary strategy would be to restore a more physiological menstrual pattern during the currently substantially prolonged ‘nubility interval’, establishing a pseudopregnancy hormonal milieu in young menstruators with a clinical diagnosis of endometriosis or adenomyosis (Eaton *et al.*, 2002).

Medically induced amenorrhoea may relieve pain, improve quality of life, and limit disease progression (Unger and Laufer, 2011), tipping the therapeutic balance in favour of this clinico-epidemiological approach (Table 2). However, prompt hormonal treatment of symptomatic young patients does not seem to constitute the standard of care. In a multicentre cross-sectional study, Pino *et al.* (2023) found that only one in five adolescents reporting symptoms highly suggestive of endometriosis were using COCs or progestogens.

**Table 2.** Pathogenic and clinical goals of menstrual suppression in the period from symptom onset to conception seeking.

1. Restore physiological amenorrhoea
2. Stop cyclic, reiterative uterine auto-traumatization
3. Limit pelvic exposure to refluxing endometrial glands
4. Reduce pelvic iron overload and oxidative stress by reducing transtubal retrograde menstruation
5. Stop repeated inflammatory events both at the endometrial–myometrial junction and on the peritoneal surface of pelvic structures
6. Decrease the oestrogenic pro-inflammatory effect and increase the progestogenic anti-inflammatory effect
7. Relieve dysmenorrhoea and improve health-related quality of life
8. Limit the potential progression of clinically diagnosed superficial peritoneal endometriosis towards infiltrating, fibrotic lesions
9. Avoid premature surgery and ovarian damage
10. Preserve reproductive potential
11. Limit the potential transition from repetitive acute pelvic pain events to chronic pelvic pain through the development of central sensitization*

* Jarrell and Arendt-Nielsen (2016a,b), Clemenza *et al.* (2021), and de Arruda *et al.* (2022).

Although surgery retains a fundamental therapeutic role in the adolescent population complaining of chronic pelvic pain, whether it should be the first-line approach is a matter of debate (Brosens *et al.*, 2013; Gordts *et al.*, 2015; Laufer and Einarsson, 2019), especially considering that (i) endometriosis would not be found in one in three severely symptomatic adolescents undergoing laparoscopy (Hirsch *et al.*, 2020; Becker *et al.*, 2022); (ii) surgery alone is not always effective, or is only partially or temporarily effective in relieving pain (Youngster *et al.*, 2013); (iii) there is no definitive evidence that early removal of endometriotic lesions as a stand-alone measure has a major impact on the natural history of the disease and on outcomes that matter to patients, i.e. likelihood of pain recurrence and future fertility (Evers, 2013); (iv) surgery removes lesions, not individual disease predisposition, and symptom and lesion recurrence are particularly common in adolescents (Tandoi *et al.*, 2011; Audebert *et al.*, 2015); (v) if long-term postoperative medical therapy should be used anyway (ACOG, 2018a; Zakhari *et al.*, 2021), whether surgery is indispensable within a strategy of medically induced amenorrhoea for years seems unclear, especially in young menstruators who respond to ovulation suppression before laparoscopy. In other words, the actual choice would not be between medical treatment or surgery, but indeed between medical treatment or surgery plus medical treatment; (vi) systematic laparoscopy in all adolescents reporting symptoms suggestive of early endometriosis implies proven, albeit limited, harms. Moreover, it would be costly (Becker *et al.*, 2022), unlikely to be cost-effective, and would consume large amounts of health care resources despite the unknown number needed to treat; (vii) acceptance of surgery as a first-line approach by symptomatic adolescents and their families would likely be limited, reducing the effectiveness of this intervention.

Unfortunately, there is no convincing evidence that early excision of limited endometriotic lesions alone, performed at a specific time during adolescence, is an effective secondary prevention measure. Therefore, surgery remains an invaluable treatment option in selected young patients who do not respond to, cannot tolerate, have contraindications to, or refuse the use of hormonal medications to suppress repetitive ovulatory menstruation (ROM), but may not be considered as an alternative to medically induced amenorrhoea in a secondary prevention strategy setting. Moreover, surgery removes endometriosis but not adenomyosis of the inner myometrium, the form most commonly observed in adolescent women (Martire *et al.*, 2023; Millischer *et al.*, 2023), whereas combined oestrogen–progestogen therapy and progestogen monotherapy suppress both diseases.

Finally, the impact of surgery on ovarian reserve in the presence of endometriomas should also be considered in the context of possible future pregnancy seeking. In addition to the follicular damage caused by the presence of an endometrioma per se, cyst removal inflicts further injury to the gonadal parenchyma. This has been demonstrated by prospectively evaluating the variation in serum anti-Müllerian hormone (AMH) levels before and after surgery and in patients with unilateral or bilateral endometriomas (Yılmaz *et al.*, 2019; Younis *et al.*, 2019). Overall, a sustained reduction in AMH values was observed following endometrioma excision, with an average drop of ∼40% when a unilateral cyst was removed and 57% when bilateral cysts were present (Younis *et al.*, 2019).

However, the use of hormonal contraceptives also causes a reduction in AMH values, which can vary between 19% and 24% for COCs (Birch Petersen *et al.*, 2015; Bernardi *et al.*, 2021; Hariton *et al.*, 2021), 22–65% for vaginal rings, and 23–27% for depot medroxyprogesterone acetate and progestogen implants (Bernardi *et al.*, 2021; Hariton *et al.*, 2021), while the hormonal IUD has little or no effect (Bernardi *et al.*, 2021; Hariton *et al.*, 2021; Nelson *et al.*, 2023). The decline in AMH with hormonal contraception does not appear to be related to cumulative duration of use (Bernardi *et al.*, 2021). Importantly, and in contrast to surgery, the suppressive effect of hormonal contraception on AMH levels is reversible (Bernardi *et al.*, 2021), and a return to normal levels has been observed within a few months of COC discontinuation (Landersoe *et al.*, 2020).

The above data provide further evidence in favour of a medical rather than a surgical first-line approach, with the aim of preventing both the formation of ovarian endometriomas and the potential need for their removal.

## The balance between potential benefits and potential harms of ROM suppression

More than 25 years ago, Brosens (1997) argued that suppression of recurrent menstrual bleeding alone should be effective in the treatment of symptomatic endometriosis, as physical elimination of all ectopic endometrial cells would be unattainable. The therapeutic goal should therefore be to prevent or suppress the recurrent bleeding associated with endometriotic lesions, with greater emphasis placed on achieving amenorrhoea than on the degree of hypo-oestrogenism induced by hormonal drugs.

This concept seems particularly relevant when choosing hormones to suppress menstruation, especially in adolescents who have not yet reached their peak bone mass. Importantly, we are here dealing with the treatment of oestrogen-dependent diseases and not just contraception. This has implications both for the hormonal content of the drugs used to induce amenorrhoea and for the trade-offs between potential benefits and potential harms to be considered in the two different clinical conditions.

Casper (2017) warns that the amount of ethinyl oestradiol (EE) even in low-dose COCs, i.e. those containing 20–30 µg of EE, is supraphysiological. This would impede adequate endometriosis control due to an excessive EE stimulatory effect that may not be effectively counteracted by the progestogens included in available COCs. Of relevance here, he argues that 5 µg of EE is equivalent to 1 mg of micronized E2 or 0.625 mg of conjugated equine oestrogens. The dose of oral conjugated oestrogen required to most closely mimic physiological mean serum oestradiol levels during the reproductive period is between 0.9 and 1.25 mg/day (Kaunitz, 2000). Based on these estimates, even the currently defined ‘low dose’ 20 µg COCs would at least double the average physiological oestrogen exposure. For this reason, Casper (2017) supports the systematic use of progestogen monotherapies rather than COCs as the first-line treatment for endometriosis.

Although this seems sensible when treating adult women who have already reached their peak bone mass, it is unclear whether such an approach is safe for adolescents, given that pharmacological secondary prevention of endometriosis and adenomyosis from clinical diagnosis until conception seeking may imply several years of ovulation suppression. In fact, it is well known that available medications can control but not eliminate lesions, and symptoms very often recur soon after drug discontinuation (Vercellini *et al.*, 2016a).

### Hormonal therapies and bone health

Almost half of adult women’s bone mass is achieved in the first few years after menarche, and bone mineral density (BMD) continues to increase after 20 years of age. Although the absolute value attained during adolescence has not yet been shown to be a reliable predictor of future pathological fracture risk (Golden, 2020; Lahoti *et al.*, 2021), limiting the optimal peak BMD by excessively and stably reducing serum oestrogen concentrations for several years in the decade between 15 and 25 years of age could reasonably be considered a risk factor for the development of postmenopausal osteoporosis (Golden, 2020).

Dienogest is currently the reference progestogen monotherapy for the treatment of endometriosis and adenomyosis (Andres *et al.*, 2015; Murji *et al.*, 2020; Kobayashi, 2023), with demonstrated antiproliferative and anti-inflammatory effects on lesions in addition to pain relief (Vannuccini *et al.*, 2018). However, the use of dienogest for 1 year in adolescents was associated with a decrease in lumbar BMD of more than 1% (Ebert *et al.*, 2017). Data on BMD changes and potential recovery after long-term use of dienogest in young women are not currently available. Therefore, Sarıdoğan (2015, 2017) warns that progestogen monotherapies may not be the optimal choice in adolescents with endometriosis.

In terms of bone loss, a very low oral dose of norethisterone acetate (NETA, 2.5 mg/day) may be a safer alternative to suppress ovulation because of the demonstrated bone-sparing effect associated with its androgenic properties and partial conversion to oestrogens (Huvinen *et al.*, 2021; American College of Obstetricians and Gynecologists’ Committee on Clinical Consensus–Gynecology, 2022; Roden, 2023). In particular, the use of NETA, 2.5 mg/day corresponds to the intake of ∼2–5 µg EE/day (Huvinen *et al.*, 2021; American College of Obstetricians and Gynecologists’ Committee on Clinical Consensus–Gynecology, 2022). However, NETA appears to be somewhat less well tolerated than dienogest, mainly due to androgenic-type cutaneous side effects and weight gain, and may induce serum lipid changes with unknown long-term effects on cardiovascular risk (Vercellini *et al.*, 2016b).

Depot medroxyprogesterone acetate should not be used for prolonged periods in adolescents, also because of consistently demonstrated significant BMD loss and increased fracture risk, which led the Food and Drug Administration to issue a black box warning almost 20 years ago. None of the available levonorgestrel-releasing intra-uterine system (LNG-IUS) (52, 19.5, and 13.5 mg) inhibit ovulation. This means that there is no adverse effect on BMD during their use, as mean serum oestrogen levels are unaffected. Data on the etonorgestrel subdermal implant in adolescents are very limited and inconclusive.

The evidence on the effect of COC use during adolescence on future bone health is inconsistent. According to a meta-analysis of nine prospective studies, the impact of COCs use in young individuals is small and the minor BMD reduction observed during medium-term follow-up seems to be of questionable clinical importance (−0.2 g/cm^2^ after 1–2 years of use) (Goshtasebi *et al.*, 2019). Of relevance, only cyclical, but not continuous, COC use seems to limit BMD gains compared to untreated adolescents (Gersten *et al.*, 2016). However, it cannot be excluded that prolonged use of COCs with EE content <20 µg, especially if a cyclical regimen is adopted, may have a negative impact on bone trophism, although definitive data on the ultimate risk of fragility fractures are not available (Golden, 2020).

It has been suggested that the transdermal delivery of oestrogens, by circumventing the liver first-pass metabolism, may prevent the adverse effects on bone health caused by the reduction in hepatic insulin-like growth factor-1 (IGF-1) synthesis induced by oral oestrogens. In fact, IGF-1 stimulates osteoblast differentiation and bone formation (for review, see Lahoti *et al.*, 2021). Thus, oestrogen-progestogen transdermal patches, and possibly vaginal rings, might be considered safe for bone health in young women. Unfortunately, there is a paucity of data on the use of these systems in adolescent populations (Di Meglio *et al.*, 2018; Lahoti *et al.*, 2021).

The long-term use of GnRH analogues, agonists and antagonists, in young girls raises concerns even when combined with add-back therapy, so that the trade-offs between such medical treatments and laparoscopy should be carefully considered in adolescents who do not respond to or cannot tolerate first-line medications (Sarıdoğan, 2015, 2017; Becker *et al.*, 2022). Indeed, one of the main adverse effects of GnRH analogues is precisely the reduction in BMD during prolonged treatment. As data on the long-term use of GnRH analogues in teenagers are insufficient, caution is warranted, and these therapies should only be chosen if they are clearly successful in patients who have failed other treatment options.

### Hormonal therapies and thromboembolic risk

The use of COCs has been consistently shown to be associated with a 3- to 5-fold increase in the risk of venous thromboembolism (VTE). However, the relative risk information should be translated into absolute risk changes to allow people understand the practical individual implications of COC use. In this regard, the baseline absolute incidence of spontaneous VTE in an average-risk adolescent population is between 4 and 11 per 100 000 women per year (Di Meglio *et al.*, 2018). The risk in young COC users varies between 10 and 30 events per 100 000 women per year (Powell, 2017). Given that the mortality rate from VTE in women aged 20–44 years is <1% (Manzoli *et al.*, 2012), in the worst-case scenario of COC use in healthy adolescents, approximately one additional death would occur per 4–500 000 young women treated annually. The baseline risk of stroke in the 15–19 age group is even lower, between 3 and 6 per 100 000 per year.

It seems fairly clear that, when considering the absolute attributable risk in adolescents without known risk factors, the excess of VTE events caused by COC use is not sufficient to offset the benefits of ovulation suppression when early-onset endometriosis and adenomyosis are diagnosed. Furthermore, patients with severe endometriosis do not appear to be at increased risk of VTE compared with the general female population of the same age (Wiegers *et al.*, 2022).

In general, the thromboembolic risk of the available COCs is determined not only by the type of progestogen (third- and fourth-generation progestogens confer a significantly higher risk than second-generation ones), but also by the type and dose of oestrogen they contain. In fact, COCs with ≥30 µg EE are associated with a higher risk of VTE than COCs with ≤20 µg (Lidegaard *et al.*, 2011; Stegeman *et al.*, 2013). In addition, the use of micronized 17β-estradiol (E2), or E2 valerate, or estetrol instead of EE appears to limit the likelihood of COCs’ side effects, including blood pressure increase, adverse serum lipid changes, and thromboembolic events (Klipping *et al.*, 2021; Chen *et al.*, 2022; Heikinheimo *et al.*, 2022; Morimont *et al.*, 2022).

The transdermal patch significantly increases EE exposure to a greater extent than a 30 µg EE containing COC (Di Meglio *et al.*, 2018), and is associated with the highest risk of VTE among available oestrogen-progestogen contraceptive combinations (Lidegaard *et al.*, 2012; Galzote *et al.*, 2017; Tepper *et al.*, 2017; Heikinheimo *et al.*, 2022). The use of the vaginal ring is also associated with an increased risk of VTE compared with the use of COCs (Lidegaard *et al.*, 2012).

Oral progestogens, subdermal implant progestogens, and the LNG-IUS are not associated with an increased risk of VTE (Lidegaard *et al.*, 2012; Heikinheimo *et al.*, 2022).

### Hormonal therapies and oncological risk

The relative risk increase for breast cancer in long-term users of COCs is ∼20–30%. However, the effect is transient and disappears a few years after COC discontinuation (Mørch *et al.*, 2017). Moreover, the average relative risk increase in the overall study population translates into very diverse absolute risk increase when COC use in different age groups is considered (Hunter, 2017). Only two excess breast cancer cases per 100 000 women younger than 35 years were observed in a large population study in Denmark (Mørch *et al.*, 2017). In a recent population-based nested case–control study (Fitzpatrick *et al.*, 2023), the 15-year absolute excess risk associated with use of COCs or progestogen-only contraceptives from the age of 16 to 20 years was 8 per 100 000 users. Whether the type and amount of oestrogen in COCs affects risk is not fully understood (Lovett *et al.*, 2017).

On the other hand, the use of COCs dramatically reduces the incidence of ovarian cancer, with a clear time-dependent response gradient. In a nationwide cohort study conducted in Denmark, the relative risk of ovarian cancer decreased from 0.82 after ≤1 year of use to 0.26 after >10 years of use. In the study population, use of hormonal contraception prevented about one in five ovarian cancers (Iversen *et al.*, 2018).

According to the very long-term results of the Royal College of General Practitioners’ Oral Contraception Study, ever-use of COCs was associated with a reduced risk of colorectal (incidence rate ratio (IRR), 0.81), endometrial (IRR, 0.66), ovarian (IRR, 0.67), and lymphoid and haematopoietic (IRR, 0.74) cancers. The increased risk of breast and cervical cancer in current and recent users disappeared after ∼5 years since COC discontinuation. In this large cohort, approximately one-third of ovarian and endometrial cancers and one-fifth of colorectal cancers were prevented by COC use. Indeed, the favourable slowdown in ovarian cancer mortality observed in Europe over the last three decades is largely due to the widespread use of COCs (Malvezzi *et al.*, 2016; Dalmartello *et al.*, 2022).

The effect of COCs on ovarian cancer risk is particularly relevant in women with endometriosis because, based on the findings of a recent meta-analysis (Kvaskoff *et al.*, 2021), their risk of this malignancy is doubled (summary relative risk, 1.93; 95% CI, 1.68–2.22). Importantly, the direction of the association between endometriosis and ovarian cancer risk can be reversed by inhibiting ovulation with COCs for several years. In fact, when Modugno *et al.* (2004) pooled information from four population-based case–control studies of incident epithelial ovarian cancer, they observed an almost 80% risk reduction in patients with a history of endometriosis who used COCs for >10 years (odds ratio, 0.21; 95% CI, 0.08–0.58).

Overall, menstruators should be informed that the net effect of long-term COC use is a small reduction in overall cancer risk (Hunter, 2017; American College of Obstetricians and Gynecologists’ Committee on Clinical Consensus–Gynecology, 2022).

## Which hormonal treatment should be preferred for suppression of repetitious ovulatory menses in young women?

When considering the use of hormones for menstrual suppression, a detailed personal and family history should be obtained and the World Health Organization’s medical eligibility criteria for contraceptive use should be applied (Altshuler *et al.*, 2015). In particular, Categories 3 (‘theoretic or proven risks usually outweigh advantages of contraceptive methods’) and 4 (‘unacceptable health risk if contraceptive method used’) preclude the use of combined oestrogen-progestogen methods. Guidelines on contraception from the Royal College of Obstetricians and Gynaecologists (RCOG), the National Institute for Health and Care Excellence (NICE), and the Faculty of Sexual and Reproductive Healthcare (FSRH) are available at https://elearning.rcgp.org.uk/mod/page/view.php?id=6961 (accessed on 24 April 2023). The U.S. Medical Eligibility Criteria for Contraceptive Use (https://www.cdc.gov/reproductivehealth/contraception/mmwr/mec/summary.html; accessed on April 24, 2023), published by the Centers for Disease Control and Prevention, may also be consulted to obtain useful information, particularly regarding relative (Category 3) or absolute (Category 4) contraindications to the use of COCs.

In young menstruators without major contraindications to oestrogen–progestogen combinations, several alternative options are available for ovulation suppression, and various factors should be considered: (i) overall, oestrogens have a prevalent pro-inflammatory effect (Straub, 2007; Cutolo *et al.*, 2014; Bulun *et al.*, 2019, 2021), whereas progestogens have an anti-inflammatory effect (Fedotcheva *et al.*, 2022); (ii) oestrogens stimulate endometriotic and adenomyotic metabolism and mitotic activity, whereas progestogens inhibit them; (iii) progestogen monotherapies are theoretically better than COCs in suppressing endometriosis, but exert a larger adverse effect on BMD compared with low-dose COCs; (iv) breakthrough bleeding and spotting during menstrual suppression are generally easier to manage with COCs than with progestogens alone; (v) the adolescent’s preference for any of the available treatment options must be given the highest priority, including the choice of surgery and the refusal of hormone therapy (Yong *et al.*, 2020).

If oestrogen-progestogen combinations are ultimately chosen, only monophasic COCs should be used for continuous, tailored regimens (Nash *et al.*, 2020). To minimize the risk of VTE, COCs containing E2 valerate or oestradiol should be preferred, and those with >20 µg EE should be avoided, also to prevent undue activation of endometriotic lesions. Moreover, E2 valerate is associated with a less pro-inflammatory effect compared with EE (Kangasniemi *et al.*, 2020).

The discontinuation rate of oestrogen-progestogen transdermal patches is particularly high in young women, probably because of detachment frequency (Powell, 2017; Lahoti *et al.*, 2021). Vaginal rings are associated with frequent spotting and breakthrough bleeding when used continuously (Vercellini *et al.*, 2010), and cannot be prescribed in adolescents before their sexual debut.

If a progestogen is preferred, oral NETA 2.5 mg/day could be the first choice, based on good efficacy, satisfactory bleeding control, bone-sparing activity, and limited cost (Kaser *et al.*, 2012; Vercellini *et al.*, 2016a,b, 2018). If NETA is not tolerated due to androgenic-type side effects, switching to oral dienogest 2 mg/day is indicated. However, due to a reduction in BMD after prolonged treatment (Ebert *et al.*, 2017; Kim *et al.*, 2021), the concomitant use of transdermal oestradiol gel, 1 mg/day, is suggested. Alternatively, an EP combination licenced in Europe for postmenopausal HRT that contains dienogest 2 mg and oestradiol valerate 1 mg, can be used with a continuous, tailored regimen.

Progestogen-only pills and the etonorgestrel 68 mg subdermal implant may not be considered a valid alternative for menstrual suppression, as their use is associated with frequent breakthrough bleeding and spotting, and amenorrhoea is achieved in only one in five women (American College of Obstetricians and Gynecologists’ Committee on Clinical Consensus–Gynecology, 2022; Edelman *et al.*, 2023).

Several authoritative international gynaecological scientific societies consistently support the use of IUDs in adolescents (AAP, 2014; Ott *et al.*, 2014; Black *et al.*, 2016; Di Meglio *et al.*, 2018; ACOG, 2018a; Margaritis *et al.*, 2023), but whether LNG-IUSs are appropriate alternatives for menstrual suppression in sexually active adolescents with endometriosis is controversial. Some experts suggest that an LNG-IUS should preferably be placed at the end of a laparoscopy to avoid the pain and discomfort likely to be experienced when inserting these devices in a young nulligravida (Sarıdoğan, 2015, 2017; Becker *et al.*, 2022).

According to the American College of Obstetricians and Gynecologists’ Committee on Clinical Consensus–Gynecology (2022), *‘For patients who may benefit from suppression of ovulation with their method of menstrual suppression, consideration should be given to the unpredictable suppression of ovulation with the LNG-IUD’.* Actually, the results of one RCT showed that an LNG-IUS was ineffective in preventing the recurrence of ovarian endometrioma after surgery (Chen *et al.*, 2017).

**Figure 2.**
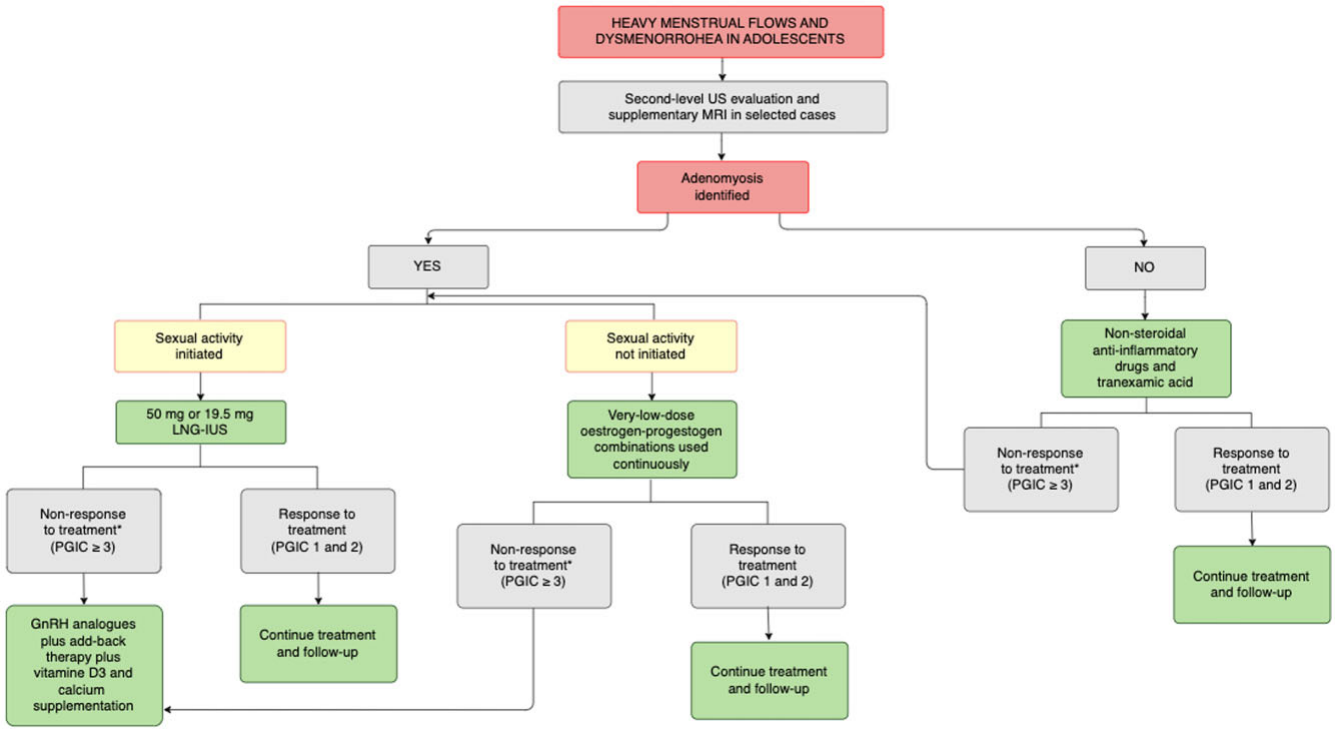
**Proposal for a diagnostic and therapeutic algorithm, including self-reported outcome measures, for the young menstruator with heavy menstrual bleeding, severe dysmenorrhoea, and a clinical suspicion of early-onset adenomyosis who accepts, tolerates, and has no contraindications to long-term hormonal menstrual suppression.** US, ultrasonographic scan; MRI, magnetic resonance imaging; LNG-IUS, levonorgestrel-releasing intra-uterine system; PGIC, patient global impression of change seven-point scale (Guy, 1976; Dworkin *et al.*, 2005). *After at least 3-month treatment.

Nevertheless, a high proportion of menstruators experience amenorrhoea a few months after insertion of the LNG-IUS, and uterine blood loss can be reduced by more than 90% due to a direct effect on the endometrium (Abbas *et al.*, 2020). Therefore, if the main complaints are dysmenorrhoea and heavy menstrual bleeding and no ovarian endometriomas or infiltrating, fibrotic lesions are found, insertion of the 52 or 19.5 mg LNG-IUS should be discussed appropriately with the young patient and her parents, taking into account the very long period of efficacy of both devices (8 and 5 years, respectively). Importantly, the LNG-IUS does not have a detrimental effect on BMD, and this is relevant when treating symptomatic adolescents. In particular, the LNG-IUS should be included among the first-line options for sexually active young women with early-onset adenomyosis detected at second-level US (Fig. 2).

GnRH analogues should be proposed as the last medical option for the shortest possible time, when all other pharmacological alternatives have failed and the young patient and her parents refuse laparoscopy. Triptorelin 3.75 mg i.m. depot preparations can be injected every 6 weeks instead of every 4 weeks (Vercellini *et al.*, 2023a), with tibolone 2.5 mg as adjunctive therapy to prevent vasomotor symptoms and BMD decline without risking reactivation of endometriotic lesions (Lindsay *et al.*, 1996; Taskin *et al.*, 1997; Castrejón-Delgado *et al.*, 2021). Vitamin D3 and calcium supplementation could be considered when prolonged treatment is planned.

GnRH antagonists are gaining momentum for the treatment of endometriosis-associated pain, and the results of several phase III RCTs have been published, showing efficacy on pain similar to that of GnRH agonists, and a safety and tolerability profile directly correlated with the degree of ovarian inhibition achieved (Yan *et al.*, 2022; Xin *et al.*, 2023). In particular, two non-peptide and orally active small molecules, i.e. elagolix and relugolix, are already marketed for the treatment of endometriosis. Combination therapy with oestradiol 1 mg and norethisterone acetate 0.5 mg, also in the same tablet, limits hypoestrogenic side effects and bone resorption and may be particularly convenient to use, thus potentially increasing adherence (Giudice *et al.*, 2022).

### Cyclic or continuous use of oestrogen-progestogen combinations?

It is now well established that the withdrawal bleeding associated with traditional COCs regimens is not physiologically necessary, was originally favoured solely for social, cultural and religious reasons, and is currently only offered with the marketing objective of increasing acceptance, compliance, and continuation by perceiving cyclical use as ‘natural’ (Kaunitz, 2000; Thomas and Ellertson, 2000; Renfree, 2012; Benson and Micks, 2015; American College of Obstetricians and Gynecologists’ Committee on Clinical Consensus–Gynecology, 2022). Furthermore, at an individual level, continued use of COCs does not result in a clinically relevant absolute increase in thromboembolic or other adverse events (Benson and Micks, 2015; MacGregor and Guillebaud, 2018).

Despite accumulating data showing that continuous use of COCs is as safe as cyclical use (Nash *et al.*, 2020), people and many doctors still hold the unfounded belief that monthly bleeding is physiological and contributes to female health, ignoring evolutionary evidence to the contrary. As a result, menstruators continue to be prescribed COCs on a cyclical basis, even though the potential health benefits of induced monthly uterine bleeding are completely unknown (Kaunitz, 2000). Indeed, there appear to be good reasons, from evolutionary, pathogenic, and clinical viewpoints, to avoid such bleeding episodes in young individuals with a clinical suspicion of endometriosis or adenomyosis (Table 2). Anticipatory counselling regarding unscheduled bleeding for patients initiating continuous COC or progestogen use is important to limit discomfort and anxiety deriving from associated pain and to provide instructions on how to manage these relatively common episodes (Zigler and McNicholas, 2017; American College of Obstetricians and Gynecologists’ Committee on Clinical Consensus–Gynecology, 2022). Women should be advised that these events are generally more frequent in the early months of treatment and tend to resolve over time.

When COCs are taken in an uninterrupted fashion, breakthrough bleeding and prolonged spotting are frequent. Therefore, the best choice for suppression of ROM in subjects with symptomatic endometriosis is the so-called tailored extended regimen (Benson and Micks, 2015) or continuous flexible regimen (MacGregor and Guillebaud, 2018), with 4- to 7-day hormone-free intervals triggered by breakthrough bleeding or prolonged spotting of ≥5 days, and followed by resumption of continuous oral contraceptive use until the next bleeding episode (Sulak *et al.*, 2006; Jensen *et al.*, 2012; Hee *et al.*, 2013; Zorbas *et al.*, 2015; Vercellini *et al.*, 2016b; MacGregor and Guillebaud, 2018; Nash *et al.*, 2020).

## Proposal of clinical algorithms for the secondary prevention of early-onset endometriosis and adenomyosis in adolescents

With the purpose of immediately suppressing ROM as soon as endometriosis or adenomyosis is suspected or diagnosed and until pregnancy is attempted, the algorithms shown in Figs 1 and 2 are proposed based on the following assumptions: (i) from an evolutionary viewpoint, ROM for decades may not be considered the physiological norm; (ii) ovulation and menstruation are inflammatory events that, if repeated unremittingly, may favour the early-onset of endometriosis and adenomyosis; (iii) the years from menarche to the first considerable rise of the incidence curve of both diseases appear crucial for lesion development; (iv) endometriosis has been observed in two-thirds of young women undergoing laparoscopy for CPP symptoms, and adenomyosis in one-fifth of adolescents complaining of heavy menstrual flow and dysmenorrhoea; (v) a non-surgical diagnosis of both conditions based on history, physical findings and US or MR imaging is valid, reliable, and has been consistently recommended (Taylor *et al.*, 2018; Agarwal *et al.*, 2019; Chapron *et al.*, 2019; Becker *et al.*, 2022).

Therefore, suppression of ROM is here intended not only as a treatment for a symptomatic condition, but primarily as a long-term, secondary-prevention interventional endocrinological measure aimed at interrupting the oestrogen-based inflammatory and fibrogenic mechanisms and, ultimately, at limiting the consequences of the mismatch between the very slow Darwinian genetic adaptation and the currently very rapid environmental (i.e. social) evolution (Table 2).

The suggested clinical algorithms are proposed only for adolescents who have no absolute contraindications to hormone therapy and who accept and tolerate pharmacologically induced amenorrhoea. The 5 cm diameter cut-off to define an ovarian endometrioma as ‘small’, for which surgery can be withheld in the absence of suspicious US features, is arbitrary and based on the proposal of Muzii *et al.* (2017). In adolescents with clinical suspicion of early-onset endometriosis (Fig. 1) who do not accept or tolerate (Yong *et al.*, 2020), or have contraindications to, hormonal therapy, laparoscopy should be performed without delay (Becker *et al.*, 2022).

The algorithms take into account the individual patient’s judgement of the outcome of medical interventions for symptom relief. As recommended by the Initiative on Methods, Measurement, and Pain Assessment in Clinical Trials (IMMPACT), we included the Patient Global Impression of Change (PGIC; Guy, 1976) as the preferred patient-reported measure to capture women’s assessment of global improvement and satisfaction with treatments for their condition (Dworkin *et al.*, 2005). The PGIC is a single-item, seven-point scale (1 = very much improved; 2 = much improved; 3 = minimally improved; 4 = no change; 5 = minimally worse; 6 = much worse; 7 = very much worse) that patients are asked to use to rate their overall condition since commencing treatment (Dworkin *et al.*, 2005). For the purposes of the proposed algorithms, we have defined the one- and two-point ratings as ‘response’ and the three- to seven-point ratings as ‘non-response’. The definition of ‘non-response’ applies after at least 3 months of unsuccessful treatment. We included the three-point rating in the ‘non-response’ category because, in our opinion and experience, a ‘minimally improved’ status may not be considered satisfactory enough to suggest continuing treatment in an adolescent complaining of severe symptoms and for whom alternative options exist.

In the algorithm for young patients with CPP symptoms and suspected early-onset endometriosis (Fig. 1), we have also included a second patient-reported outcome measure, the Central Sensitization Inventory (CSI), a questionnaire designed to identify those patients whose pain is complicated by CNS sensitization (Mayer *et al.*, 2012) and who may not respond optimally to conventional treatments (Cuesta-Vargas *et al.*, 2020; Cetera *et al.*, 2023b). The CSI has been validated in both the general chronic pain population (Cuesta-Vargas *et al.*, 2018; Scerbo *et al.*, 2018) and in women with endometriosis (Orr *et al.*, 2020, 2022; Raimondo *et al.*, 2023; Cetera *et al.*, 2023a). The CSI part A consists of 25 questions, and patients are asked to rate each question from 0 to 4 (0 = never; 1 = rarely; 2 = sometimes; 3 = often; 4 = always) for a total maximum score of 100. Neblett *et al.* (2017) established the following CSI severity levels: 0–29 points = subclinical; 30–39 points = mild; 40–49 = moderate; 50–59 = severe; 60–100 = extreme. A CSI part A cut-off score of 40 points has a sensitivity of 78% and a specificity of 80% for detecting women with endometriosis and ≥3 co-existing central sensitization syndromes (Orr *et al.*, 2022).

A ≥40 CSI score can be used to screen those endometriosis patients in whom central sensitization mechanisms are likely to be involved in determining the overall pain experience (Orr *et al.*, 2020, 2022). In addition, it was observed that individuals with higher baseline CSI scores had, as expected, worse follow-up outcomes after surgery for symptomatic endometriosis (Orr *et al.*, 2023b). Given the very high prevalence (∼50%) of a CSI score ≥40 in women with endometriosis (Orr *et al.*, 2022; Raimondo *et al.*, 2023), we indicated a cut-off score range between ≥40 and ≥60 in the algorithm for early-onset endometriosis (Fig. 1). Thus, patients with moderate, severe, or only extreme central sensitization levels may be considered according to local protocols (Cetera *et al.*, 2023a). Translated and psychometrically validated CSI versions in different languages can be found at https://www.pridedallas.com/questionnaires.

The addition of a GnRH agonist test solely in non-responders to first-line therapy and in the absence of USA and MRI evidence of ovarian and infiltrating endometriosis, is based on the assumption that lack of improvement after 3 months of profound hypo-oestrogenism substantially reduces the likelihood that superficial peritoneal disease is the cause of pain symptoms. Although a response to the GnRH agonist test is not definitive proof of the presence of endometriosis, it increases the likelihood enough to consider that laparoscopy may be reasonably indicated (Practice Committee of American Society for Reproductive Medicine, 2008; Howard, 2009; Vercellini *et al.*, 2014). Laparoscopy has been included in the final part of the algorithm only, unless patients specifically request it early in the diagnostic work-up, also because the therapeutic value of this surgical procedure in the case of otherwise unidentifiable superficial peritoneal implants has recently been questioned (Chapron *et al.*, 2019; Horne *et al.*, 2019; Becker *et al.*, 2022; Tucker *et al.*, 2023).

The recommendation for the use of postoperative medical therapy is based on the results of a recent systematic review and meta-analysis, which showed an impressive reduction in the risk of pain and lesion recurrence after surgery for symptomatic endometriosis in patients on long-term suppressive therapy compared with those on expectant management alone (Zakhari *et al.*, 2021).

The above algorithms are amenable to even substantial changes as soon as relevant scientific information regarding the pathogenesis, diagnosis, and treatment of endometriosis and adenomyosis becomes available.

## Prospectus: raising awareness among physicians, informing families, counselling adolescents

In young women, the normalization and dismissal of menstrual pain and heavy menstrual flow, and the minimization of non-menstrual, acyclic abdominal pain by parents, relatives, teachers, and peers, combined with limited medical awareness of the possible organic causes of the reported symptoms, can be extremely detrimental to health-related quality of life in all its aspects (Ng *et al.*, 2020b; Cetera *et al.*, 2023b), with possible consequences for the progression of endometriosis and adenomyosis and impairment of reproductive potential.

In addition, when considering ovulation suppression in general and in adolescents in particular, it is sometimes unclear whether it is the clinician or the patient and her family who find it more difficult to overcome false myths. Ideally, both parties should be aware that, in the post-menarchal decade, ROM is not necessarily physiological. Understanding this concept is even more important when teenagers present with symptoms suggestive of early-onset endometriosis and adenomyosis, as prejudices must not interfere with a correct clinical diagnosis or delay prompt preventive interventional endocrinological measures. Young women (and their general practitioners, paediatricians, gynaecologists, and school teachers also) should be reassured that hormonal suppression of ROM does not affect ovarian function, which resumes soon after drug discontinuation; does not impair future fertility; does not increase the overall risk of cancer; has clinically irrelevant effects on the individual likelihood of VTE, provided that recognized major risk factors are excluded; and does not in itself lead to significant weight gain.

It should be tactfully explained that ovulation suppression should not be considered an anomaly and that it can be useful not only for symptom relief but also for the prevention of future benign and malignant gynaecological conditions. It should also be explained, in simple terms that lay people can understand, that interrupting repetitive cyclic acute painful events can reduce the risk of developing central sensitization and overlapping chronic pain conditions (Jarrell and Arendt-Nielsen, 2016a,b; de Arruda *et al.*, 2022).

If ovulation suppression is ultimately chosen, the adolescent and parents should be informed that amenorrhoea may not be achieved easily and immediately (American College of Obstetricians and Gynecologists’ Committee on Clinical Consensus–Gynecology, 2022). The occurrence of irregular bleeding should be anticipated to avoid unpreparedness and undue anxiety, instructions given on how to manage these events (e.g. tailored cycling), and reassurance given about the generally decreasing frequency of bleeding episodes over time.

In adolescents with suspected severe (≥50 points) or extreme (≥60 points) central sensitization based on the CSI score and no clinical or imaging evidence of endometriotic lesions, the indication for laparoscopy should be considered with caution. Young patients and their parents need to be informed that, in these conditions, the likelihood of successful and sustained pain relief may be reduced (Tucker *et al.*, 2023; Orr *et al.*, 2023b), and that in a small proportion of cases pain may even worsen (Horne *et al.*, 2019). Informed consent should be tailored to this particular patient profile, avoiding the use of standard forms. A multidisciplinary CPP assessment should be proposed, and non-pharmacological interventions tried before a final decision is made to proceed with surgery.

Healthcare providers should be gentle and clear, offer complete and understandable information, be sensitive and empathetic, describe in detail all available treatment options with their potential benefits and harms, always interact simultaneously with both the adolescent and her parents, give ample opportunity to express fears and doubts, and respect the young woman’s preferences and priorities. This would promote a truly shared decision-making process, confirming trust in the doctor and preventing the negative perception of the medical profession that can arise in endometriosis patients when they receive suboptimal care (Ng *et al.*, 2020b), and facilitate treatment acceptance and adherence, thus optimizing effectiveness.

## Preventing sclerosing endometriosis and adenomyosis: mission impossible?

We disclose our collective intellectual conflict of interest regarding the role of surgery in the management of endometriosis, which we believe arrives too late when lesions are already established. With regard to adenomyosis, surgery is generally considered cumbersome, often not radical, and with unpredictable effects. Moreover, early-onset adenomyosis in young women usually affects the sub-endometrial part of the inner myometrium, making excisional treatments impossible.

This opinion article has several limitations, which are listed in Part I (Vercellini *et al.*, 2023b). Briefly, the relevant literature was reviewed comprehensively but not systematically, therefore some important studies may have been overlooked, or we cannot exclude that our intellectual competing interest may have led to selective referencing. In addition, the quality of the included studies was not formally assessed.

In addition, there is currently no evidence that early and sustained suppression of ROM improves long-term clinical outcomes, particularly fertility preservation, and it is questionable to promote extended preventive hormonal intervention on the basis of data on pathogenesis derived only from cross-sectional and case–control studies. Protracted cohort studies would be needed to justify such a demanding approach. What may be desirable in endometriosis research are prolonged, prospective observational studies, that follow the natural history of symptoms and lesions from adolescence to adulthood. Similar studies have been conducted in the field of reproductive endocrinology (e.g. the transition from premenopause to menopause; the Melbourne Women’s Midlife Health Project study) (Guthrie *et al.*, 2004) and obstetrics (e.g. the effect of stressful events during pregnancy on future events in the offspring; the Raine study) (Straker *et al.*, 2015).

However, our aim was to raise awareness of the current unphysiological postmenarchal menstrual pattern and to stimulate debate about the potentially related pathogenic downstream consequences, not to provide definitive evidence of the validity of our construct, as this would require decades of future research, including not only prospective observational studies but also intervention studies to assess treatment outcomes (Shah and Missmer, 2011). Moreover, the potential benefits of medically induced amenorrhoea, including pain relief and normalization of health-related quality of life, are important anyway, whereas the potential harms, including VTE and breast cancer, are extremely rare at such a young age. Achievement of peak bone mass may be an issue, and monitoring BMD in young women during prolonged treatment may be considered. For those adolescents that cannot tolerate the side effects associated with first-line hormone therapy, such as intractable irregular bleeding, depressed mood, and decreased libido, surgery remains an alternative option.

Jarrell and Arendt-Nielsen (2016a) suggest that a broader evolutionary perspective, including the notion of a maladaptive status between biological and cultural evolution leading to recurrent dysmenorrhoea, could modify the concept of what constitutes a normal menstrual pattern, and potentially promote prevention and treatment studies that rely on menstrual suppression also.

Indeed, the future health and reproductive potential of many young menstruators is at stake. This may tip the balance in favour of suppression of ROM, regardless of the actual aetiology of both endometriosis and adenomyosis, as this seems the most prudent course of action. It is also urgent to verify whether timely prevention of lesion progression could have an impact on some severe and increasingly common obstetrical complications associated with advanced forms of endometriosis and adenomyosis (Mandelbaum *et al.*, 2023; Park *et al.*, 2023; Vercellini *et al.*, 2023c).

In 1976, Roger Valentine Short wrote ‘*we should also try to recapture what civilization has destroyed, the ability to keep the ovaries and the female reproductive tract in a state of quiescence when reproduction is not desired. Women may be physiologically ill-adapted to spend the greater part of their reproductive lives having an endless succession of menstrual cycles*’ (Short, 1976). After almost half a century, perhaps it is time to consider whether his hypothesis is worth testing.

## Data Availability

The data included in this article were extracted as published in the available original articles. No new data were generated or analysed to support this paper.

## References

[dead206-B1] Abbas AM , Samy A, Atwa K, Ghoneim HM, Lotfy M, Saber Mohammed H, Abdellah AM, El Bahie AM, Aboelroose AA, El Gedawy AM et al The role of levonorgestrel intra-uterine system in the management of adenomyosis: a systematic review and meta-analysis of prospective studies. Acta Obstet Gynecol Scand 2020;99:571–581. 31889294 10.1111/aogs.13798

[dead206-B2] ACOG. ACOG Committee Opinion No. 735: adolescents and long-acting reversible contraception: implants and intrauterine devices. Obstet Gynecol2018a;131:e130–e139. 29683910 10.1097/AOG.0000000000002632

[dead206-B3] ACOG. ACOG Committee Opinion No. 760: dysmenorrhea and endometriosis in the adolescent. Obstet Gynecol2018b;132:e249–e258. 30461694 10.1097/AOG.0000000000002978

[dead206-B4] Agarwal SK , Chapron C, Giudice LC, Laufer MR, Leyland N, Missmer SA, Singh SS, Taylor HS. Clinical diagnosis of endometriosis: a call to action. Am J Obstet Gynecol 2019;220:354.e1–354.e12. 10.1016/j.ajog.2018.12.039 30625295

[dead206-B5] Allaire C , Bedaiwy MA, Yong PJ. Diagnosis and management of endometriosis. CMAJ 2023;195:E363–E371. 36918177 10.1503/cmaj.220637 PMC10120420

[dead206-B6] Altshuler AL , Gaffield ME, Kiarie JN. The WHO's medical eligibility criteria for contraceptive use: 20 years of global guidance. Curr Opin Obstet Gynecol 2015;27:451–459. 26390246 10.1097/GCO.0000000000000212 PMC5703409

[dead206-B8] American Academy of Pediatrics (AAP). Contraception for Adolescents. Pediatrics 2014;134:e1244–e1256. 25266430 10.1542/peds.2014-2299

[dead206-B7] American College of Obstetricians and Gynecologists’ Committee on Clinical Consensus–Gynecology. General Approaches to Medical Management of Menstrual Suppression: ACOG Clinical Consensus No. 3. Obstet Gynecol 2022;140:528–541. 36356248 10.1097/AOG.0000000000004899

[dead206-B9] Andres MdP , Lopes LA, Baracat EC, Podgaec S. Dienogest in the treatment of endometriosis: systematic review. Arch Gynecol Obstet 2015;292:523–529. 25749349 10.1007/s00404-015-3681-6

[dead206-B10] Anglesio MS , Papadopoulos N, Ayhan A, Nazeran TM, Noë M, Horlings HM, Lum A, Jones S, Senz J, Seckin T et al Cancer-associated mutations in endometriosis without cancer. N Engl J Med 2017;376:1835–1848. 28489996 10.1056/NEJMoa1614814 PMC5555376

[dead206-B11] Audebert A , Lecointre L, Afors K, Koch A, Wattiez A, Akladios C. Adolescent endometriosis: report of a series of 55 cases with a focus on clinical presentation and long-term issues. J Minim Invasive Gynecol 2015;22:834–840. 25850071 10.1016/j.jmig.2015.04.001

[dead206-B12] Bartolacci C , Andreani C, Vale G, Berto S, Melegari M, Crouch AC, Baluya DL, Kemble G, Hodges K, Starrett J et al Targeting de novo lipogenesis and the Lands cycle induces ferroptosis in KRAS-mutant lung cancer. Nat Commun 2022;13:4327. 35882862 10.1038/s41467-022-31963-4 PMC9325712

[dead206-B13] Becker CM , Bokor A, Heikinheimo O, Horne A, Jansen F, Kiesel L, King K, Kvaskoff M, Nap A, Petersen K et al; ESHRE Endometriosis Guideline Group. ESHRE guideline: endometriosis. Hum Reprod Open 2022;2022:hoac009. 35350465 10.1093/hropen/hoac009 PMC8951218

[dead206-B14] Benson LS , Micks EA. Why stop now? Extended and continuous regimens of combined hormonal contraceptive methods. Obstet Gynecol Clin North Am 2015;42:669–681. 26598308 10.1016/j.ogc.2015.07.009

[dead206-B15] Bernardi LA , Weiss MS, Waldo A, Harmon Q, Carnethon MR, Baird DD, Wise LA, Marsh EE. Duration, recency, and type of hormonal contraceptive use and antimüllerian hormone levels. Fertil Steril 2021;116:208–217. 33752880 10.1016/j.fertnstert.2021.02.007 PMC8217153

[dead206-B16] Birch Petersen K , Hvidman HW, Forman JL, Pinborg A, Larsen EC, Macklon KT, Sylvest R, Andersen AN. Ovarian reserve assessment in users of oral contraception seeking fertility advice on their reproductive lifespan. Hum Reprod 2015;30:2364–2375. 26311148 10.1093/humrep/dev197

[dead206-B17] Black A , Guilbert E, Costescu D, Dunn S, Fisher W, Kives S, Mirosh M, Norman WV, Pymar H, Reid R et al Canadian contraception consensus (part 3 of 4): Chapter 7—Intrauterine contraception. J Obstet Gynaecol Can 2016;38:182–222. 27032746 10.1016/j.jogc.2015.12.002

[dead206-B18] Brosens IA. Endometriosis–a disease because it is characterized by bleeding. Am J Obstet Gynecol 1997;176:263–267. 9065165 10.1016/s0002-9378(97)70482-4

[dead206-B19] Brosens I , Gordts S, Benagiano G. Endometriosis in adolescents is a hidden, progressive and severe disease that deserves attention, not just compassion. Hum Reprod 2013;28:2026–2031. 23739215 10.1093/humrep/det243 PMC3712662

[dead206-B20] Bulun SE. Endometriosis caused by retrograde menstruation: now demonstrated by DNA evidence. Fertil Steril 2022;118:535–536. 36116802 10.1016/j.fertnstert.2022.07.012

[dead206-B21] Bulun SE , Yildiz S, Adli M, Chakravarti D, Parker JB, Milad M, Yang L, Chaudhari A, Tsai S, Wei JJ et al Endometriosis and adenomyosis: shared pathophysiology. Fertil Steril 2023;119:746–750. 36925057 10.1016/j.fertnstert.2023.03.006

[dead206-B22] Bulun SE , Yildiz S, Adli M, Wei JJ. Adenomyosis pathogenesis: insights from next-generation sequencing. Hum Reprod Update 2021;27:1086–1097. 34131719 10.1093/humupd/dmab017 PMC8543024

[dead206-B23] Bulun SE , Yilmaz BD, Sison C, Miyazaki K, Bernardi L, Liu S, Kohlmeier A, Yin P, Milad M, Wei J. Endometriosis. Endocr Rev 2019;40:1048–1079. 30994890 10.1210/er.2018-00242 PMC6693056

[dead206-B24] Casper RF. Progestin-only pills may be a better first-line treatment for endometriosis than combined estrogen-progestin contraceptive pills. Fertil Steril 2017;107:533–536. 28162779 10.1016/j.fertnstert.2017.01.003

[dead206-B25] Castrejón-Delgado L , Castelán-Martínez OD, Clark P, Garduño-Espinosa J, Mendoza-Núñez VM, Sánchez-Rodríguez MA. Effect of tibolone on bone mineral density in postmenopausal women: systematic review and meta-analysis. Biology (Basel) 2021;10:211. 33802101 10.3390/biology10030211 PMC8000366

[dead206-B26] Cetera GE , Merli CEM, Barbara G, Caia C, Vercellini P. Questionnaires for the assessment of central sensitization in endometriosis: what is the available evidence? A systematic review with a narrative synthesis. *Reprod Sci*2023a. 10.1007/s43032-023-01343-4. PMC10912156 37751146

[dead206-B27] Cetera GE , Merli CEM, Facchin F, Viganò P, Pesce E, Caprara F, Vercellini P. Non-response to first-line hormonal treatment for symptomatic endometriosis: overcoming tunnel vision. A narrative review. BMC Womens Health2023b;23:347. 37391793 10.1186/s12905-023-02490-1 PMC10311799

[dead206-B28] Chapron C , Marcellin L, Borghese B, Santulli P. Rethinking mechanisms, diagnosis and management of endometriosis. Nat Rev Endocrinol 2019;15:666–682. 31488888 10.1038/s41574-019-0245-z

[dead206-B29] Chen MJ , Jensen JT, Kaunitz AM, Achilles SL, Zatik J, Weyers S, Piltonen T, Suturina L, Apolikhina I, Bouchard C et al Tolerability and safety of the estetrol/drospirenone combined oral contraceptive: pooled analysis of two multicenter, open-label phase 3 trials. Contraception 2022;116:44–50. 36257374 10.1016/j.contraception.2022.10.004

[dead206-B30] Chen YJ , Hsu TF, Huang BS, Tsai HW, Chang YH, Wang PH. Postoperative maintenance levonorgestrel-releasing intrauterine system and endometrioma recurrence: a randomized controlled study. Am J Obstet Gynecol 2017;216:582.e1–582.e9. 10.1016/j.ajog.2017.02.008 28209488

[dead206-B31] Clemenza S , Vannuccini S, Capezzuoli T, Meleca CI, Pampaloni F, Petraglia F. Is primary dysmenorrhea a precursor of future endometriosis development? Gynecol Endocrinol 2021;37:287–293. 33569996 10.1080/09513590.2021.1878134

[dead206-B32] Cuesta-Vargas AI , Neblett R, Chiarotto A, Kregel J, Nijs J, van Wilgen CP, Pitance L, Knezevic A, Gatchel RJ, Mayer TG et al Dimensionality and reliability of the central sensitization inventory in a pooled multicountry sample. J Pain 2018;19:317–329. 29198933 10.1016/j.jpain.2017.11.006

[dead206-B33] Cuesta-Vargas AI , Neblett R, Nijs J, Chiarotto A, Kregel J, van Wilgen CP, Pitance L, Knezevic A, Gatchel RJ, Mayer TG et al Establishing central sensitization-related symptom severity subgroups: a multicountry study using the central sensitization inventory. Pain Med 2020;21:2430–2440. 33118603 10.1093/pm/pnaa210

[dead206-B34] Cutolo M , Sulli A, Straub RH. Estrogen’s effects in chronic autoimmune/inflammatory diseases and progression to cancer. Expert Rev Clin Immunol 2014;10:31–39. 24308839 10.1586/1744666X.2014.863149

[dead206-B35] Dalmartello M , La Vecchia C, Bertuccio P, Boffetta P, Levi F, Negri E, Malvezzi M. European cancer mortality predictions for the year 2022 with focus on ovarian cancer. Ann Oncol 2022;33:330–339. 35090748 10.1016/j.annonc.2021.12.007

[dead206-B36] D’Arpe S , Di Feliciantonio M, Candelieri M, Franceschetti S, Piccioni MG, Bastianelli C. Ovarian function during hormonal contraception assessed by endocrine and sonographic markers: a systematic review. Reprod Biomed Online 2016;33:436–448. 27527655 10.1016/j.rbmo.2016.07.010

[dead206-B37] de Arruda GT , Driusso P, Rodrigues JC, de Godoy AG, Degani A, Danna-Dos-Santos A, Avila MA. Are menstrual symptoms associated with central sensitization inventory? A cross-sectional study. Eur J Pain 2022;26:1759–1767. 35761773 10.1002/ejp.1999

[dead206-B38] D’Hooghe TM , Debrock S. Endometriosis, retrograde menstruation and peritoneal inflammation in women and in baboons. Hum Reprod Update 2002;8:84–88. 11866244 10.1093/humupd/8.1.84

[dead206-B39] Di Meglio G , Crowther C, Simms J. Contractive care for Canadian youth. Paediatr Child Health 2018;23:271–277. 30681670 10.1093/pch/pxx192 PMC6007342

[dead206-B40] DiVasta AD , Vitonis AF, Laufer MR, Missmer SA. Spectrum of symptoms in women diagnosed with endometriosis during adolescence vs adulthood. Am J Obstet Gynecol 2018;218:324.e1–324.e11. 10.1016/j.ajog.2017.12.007 29247637

[dead206-B41] DiVasta AD , Zimmerman LA, Vitonis AF, Fadayomi AB, Missmer SA. Overlap between irritable bowel syndrome diagnosis and endometriosis in adolescents. Clin Gastroenterol Hepatol 2021;19:528–537.e1. 32184183 10.1016/j.cgh.2020.03.014

[dead206-B42] Donnez J , Binda MM, Donnez O, Dolmans MM. Oxidative stress in the pelvic cavity and its role in the pathogenesis of endometriosis. Fertil Steril 2016;106:1011–1017. 27521769 10.1016/j.fertnstert.2016.07.1075

[dead206-B43] Dworkin RH , Turk DC, Farrar JT, Haythornthwaite JA, Jensen MP, Katz NP, Kerns RD, Stucki G, Allen RR, Bellamy N et al; IMMPACT. Core outcome measures for chronic pain clinical trials: IMMPACT recommendations. Pain 2005;113:9–19. 15621359 10.1016/j.pain.2004.09.012

[dead206-B44] Eaton SB , Strassman BI, Nesse RM, Neel JV, Ewald PW, Williams GC, Weder AB, Eaton SB, Lindeberg S, Konner MJ et al Evolutionary health promotion. Prev Med 2002;34:109–118. 11817903 10.1006/pmed.2001.0876

[dead206-B45] Ebert AD , Dong L, Merz M, Kirsch B, Francuski M, Böttcher B, Roman H, Suvitie P, Hlavackova O, Gude K et al Dienogest 2 mg daily in the treatment of adolescents with clinically suspected endometriosis: the VISanne Study to Assess Safety in ADOlescents. J Pediatr Adolesc Gynecol 2017;30:560–567. 28189702 10.1016/j.jpag.2017.01.014

[dead206-B46] Edelman A , Boniface E, Schrote K, Messerle-Forbes M, O'Donnell A, Jensen JT, Han L. Treatment of unfavorable bleeding patterns in contraceptive implant users: a randomized clinical trial of curcumin. Am J Obstet Gynecol 2023;229:145.e1–145.e9. 10.1016/j.ajog.2023.04.028 37116825

[dead206-B47] ESHRE Capri Workshop Group. Ovarian and endometrial function during hormonal contraception. Hum Reprod 2001;16:1527–1535. 11425842 10.1093/humrep/16.7.1527

[dead206-B48] Evers JL. Is adolescent endometriosis a progressive disease that needs to be diagnosed and treated? HumReprod 2013;28:2023. 10.1093/humrep/det298 23861497

[dead206-B49] Exacoustos C , Lazzeri L, Martire FG, Russo C, Martone S, Centini G, Piccione E, Zupi E. Ultrasound findings of adenomyosis in adolescents: type and grade of the disease. J Minim Invasive Gynecol 2022;29:291–299.e1. 34464760 10.1016/j.jmig.2021.08.023

[dead206-B50] Fedotcheva TA , Fedotcheva NI, Shimanovsky NL. Progesterone as an anti-inflammatory drug and immunomodulator: new aspects in hormonal regulation of the inflammation. Biomolecules 2022;12:1299. 36139138 10.3390/biom12091299 PMC9496164

[dead206-B51] Fitzpatrick D , Pirie K, Reeves G, Green J, Beral V. Combined and progestagen-only hormonal contraceptives and breast cancer risk: a UK nested case-control study and meta-analysis. PLoS Med 2023;20:e1004188. 36943819 10.1371/journal.pmed.1004188 PMC10030023

[dead206-B52] Galzote RM , Rafie S, Teal R, Mody SK. Transdermal delivery of combined hormonal contraception: a review of the current literature. Int J Womens Health 2017;9:315–321. 28553144 10.2147/IJWH.S102306 PMC5440026

[dead206-B53] Gersten J , Hsieh J, Weiss H, Ricciotti NA. Effect of extended 30 μg ethinyl estradiol with continuous low-dose ethinyl estradiol and cyclic 20 μg ethinyl estradiol oral contraception on adolescent bone density: a randomized trial. J Pediatr Adolesc Gynecol 2016;29:635–642. 27287084 10.1016/j.jpag.2016.05.012

[dead206-B54] Geysenbergh B , Dancet EAF, D'Hooghe T. Detecting endometriosis in adolescents: why not start from self-report screening questionnaires for adult women? Gynecol Obstet Invest 2017;82:322–328. 27816976 10.1159/000452098

[dead206-B55] Giudice LC , As-Sanie S, Arjona Ferreira JC, Becker CM, Abrao MS, Lessey BA, Brown E, Dynowski K, Wilk K, Li Y et al Once daily oral relugolix combination therapy versus placebo in patients with endometriosis-associated pain: two replicate phase 3, randomised, double-blind, studies (SPIRIT 1 and 2). Lancet 2022;399:2267–2279. 35717987 10.1016/S0140-6736(22)00622-5

[dead206-B56] Golden NH. Bones and birth control in adolescent girls. J Pediatr Adolesc Gynecol 2020;33:249–254. 31972296 10.1016/j.jpag.2020.01.003

[dead206-B57] Gordts S , Puttemans P, Gordts S, Brosens I. Ovarian endometrioma in the adolescent: a plea for early-stage diagnosis and full surgical treatment. Gynecol Surg 2015;12:21–30. 25774119 10.1007/s10397-014-0877-x PMC4349957

[dead206-B58] Goshtasebi A , Subotic Brajic T, Scholes D, Beres Lederer Goldberg T, Berenson A, Prior JC. Adolescent use of combined hormonal contraception and peak bone mineral density accrual: a meta-analysis of international prospective controlled studies. Clin Endocrinol (Oxf) 2019;90:517–524. 30614555 10.1111/cen.13932 PMC6850432

[dead206-B59] Guo SW , Habiba M, Benagiano G. From retrograde menstruation to endometrial determinism and a brave new world of “root treatment” of endometriosis: destiny or a fanciful utopia? Biomolecules 2023;13:336. 36830705 10.3390/biom13020336 PMC9953699

[dead206-B60] Guthrie JR , Dennerstein L, Taffe JR, Lehert P, Burger HG. The menopausal transition: a 9-year prospective population-based study. The Melbourne Women’s Midlife Health Project. Climacteric 2004;7:375–389. 15799609 10.1080/13697130400012163

[dead206-B61] Guy W. ECDEU Assessment Manual for Psychopharmacology (DHEW Publication No. ADM 76-338). Washington, DC: US Government Printing Office, 1976.

[dead206-B62] Hariton E , Shirazi TN, Douglas NC, Hershlag A, Briggs SF. Anti-Müllerian hormone levels among contraceptive users: evidence from a cross-sectional cohort of 27,125 individuals. Am J Obstet Gynecol 2021;225:515.e1–515.e10. 10.1016/j.ajog.2021.06.052 34126087

[dead206-B63] Hee L , Kettner LO, Vejtorp M. Continuous use of oral contraceptives: an overview of effects and side-effects. Acta Obstet Gynecol Scand 2013;92:125–136. 23083413 10.1111/aogs.12036

[dead206-B64] Heikinheimo O , Toffol E, Partonen T, But A, Latvala A, Haukka J. Systemic hormonal contraception and risk of venous thromboembolism. Acta Obstet Gynecol Scand 2022;101:846–855. 35633036 10.1111/aogs.14384 PMC9564731

[dead206-B65] Hirsch M , Dhillon-Smith R, Cutner AS, Yap M, Creighton SM. The prevalence of endometriosis in adolescents with pelvic pain: a systematic review. J Pediatr Adolesc Gynecol 2020;33:623–630. 32736134 10.1016/j.jpag.2020.07.011

[dead206-B66] Hoppenbrouwers K , Roelants M, Meuleman C, Rijkers A, Van Leeuwen K, Desoete A, D'Hooghe T. Characteristics of the menstrual cycle in 13-year-old Flemish girls and the impact of menstrual symptoms on social life. Eur J Pediatr 2016;175:623–630. 26670027 10.1007/s00431-015-2681-7

[dead206-B67] Horne AW , Daniels J, Hummelshoj L, Cox E, Cooper KG. Surgical removal of superficial peritoneal endometriosis for managing women with chronic pelvic pain: time for a rethink? BJOG 2019;126:1414–1416. 31359584 10.1111/1471-0528.15894 PMC6852286

[dead206-B68] Horne AW , Missmer SA. Pathophysiology, diagnosis, and management of endometriosis. BMJ 2022;379:e070750. 36375827 10.1136/bmj-2022-070750

[dead206-B69] Howard FM. Endometriosis and mechanisms of pelvic pain. J Minim Invasive Gynecol 2009;16:540–550. 19835795 10.1016/j.jmig.2009.06.017

[dead206-B70] Hunter DJ. Oral contraceptives and the small increased risk of breast cancer. N Engl J Med 2017;377:2276–2277. 29211666 10.1056/NEJMe1709636

[dead206-B71] Huvinen E , Holopainen E, Heikinheimo O. Norethisterone and its acetate—what’s so special about them? BMJ Sex Reprod Health 2021;47:102–109. 10.1136/bmjsrh-2020-200619 32398290

[dead206-B72] Inoue S , Hirota Y, Ueno T, Fukui Y, Yoshida E, Hayashi T, Kojima S, Takeyama R, Hashimoto T, Kiyono T et al Uterine adenomyosis is an oligoclonal disorder associated with KRAS mutations. Nat Commun 2019;10:5785. 31857578 10.1038/s41467-019-13708-y PMC6923389

[dead206-B73] Iversen L , Fielding S, Lidegaard Ø, Mørch LS, Skovlund CW, Hannaford PC. Association between contemporary hormonal contraception and ovarian cancer in women of reproductive age in Denmark: prospective, nationwide cohort study. BMJ 2018;362:k3609. 30257920 10.1136/bmj.k3609 PMC6283376

[dead206-B74] Jarrell J , Arendt-Nielsen L. Allodynia and dysmenorrhea. J Obstet Gynaecol Can2016a;38:270–274. 27106198 10.1016/j.jogc.2016.02.001

[dead206-B75] Jarrell J , Arendt-Nielsen L. Evolutionary considerations in the development of chronic pelvic pain. Am J Obstet Gynecol2016b;215:201.e1–201.e4. 10.1016/j.ajog.2016.05.019 27269450

[dead206-B76] Jensen JT , Garie SG, Trummer D, Elliesen J. Bleeding profile of a flexible extended regimen of ethinylestradiol/drospirenone in US women: an open-label, three-arm, active-controlled, multicenter study. Contraception 2012;86:110–118. 22281416 10.1016/j.contraception.2011.12.009

[dead206-B77] Kangasniemi MH , Haverinen A, Luiro K, Hiltunen JK, Komsi EK, Arffman RK, Heikinheimo O, Tapanainen JS, Piltonen TT. Estradiol valerate in COC has more favorable inflammatory profile than synthetic ethinyl estradiol: a randomized trial. J Clin Endocrinol Metab 2020;105:dgaa186. 32303765 10.1210/clinem/dgaa186

[dead206-B78] Kaser DJ , Missmer SA, Berry KF, Laufer MR. Use of norethindrone acetate alone for postoperative suppression of endometriosis symptoms. J Pediatr Adolesc Gynecol 2012;25:105–108. 22154396 10.1016/j.jpag.2011.09.013

[dead206-B79] Kaunitz AM. Menstruation: choosing whether…and when. Contraception 2000;62:277–284. 11239613 10.1016/s0010-7824(00)00182-7

[dead206-B80] Kim SE , Lim HH, Lee DY, Choi D. The long-term effect of dienogest on bone mineral density after surgical treatment of endometrioma. Reprod Sci 2021;28:1556–1562. 33449347 10.1007/s43032-020-00453-7

[dead206-B81] Klipping C , Duijkers I, Mawet M, Maillard C, Bastidas A, Jost M, Foidart JM. Endocrine and metabolic effects of an oral contraceptive containing estetrol and drospirenone. Contraception 2021;103:213–221. 33428907 10.1016/j.contraception.2021.01.001

[dead206-B82] Kobayashi H. Efficacy, adverse events, and challenges of dienogest in the management of symptomatic adenomyosis: a comparison with different hormonal treatments. Gynecol Obstet Invest 2023;88:71–80. 36682346 10.1159/000529185

[dead206-B83] Kvaskoff M , Bijon A, Clavel-Chapelon F, Mesrine S, Boutron-Ruault MC. Childhood and adolescent exposures and the risk of endometriosis. Epidemiology 2013;24:261–269. 23337239 10.1097/EDE.0b013e3182806445

[dead206-B84] Kvaskoff M , Mahamat-Saleh Y, Farland LV, Shigesi N, Terry KL, Harris HR, Roman H, Becker CM, As-Sanie S, Zondervan KT et al Endometriosis and cancer: a systematic review and meta-analysis. Hum Reprod Update 2021;27:393–420. 33202017 10.1093/humupd/dmaa045

[dead206-B85] Laganà AS , Vitale SG, Muscia V, Rossetti P, Buscema M, Triolo O, Rapisarda AM, Giunta L, Palmara V, Granese R et al Endometrial preparation with dienogest before hysteroscopic surgery: a systematic review. Arch Gynecol Obstet 2017;295:661–667. 27904953 10.1007/s00404-016-4244-1

[dead206-B86] Lahoti A , Yu C, Brar PC, Dalgo A, Gourgari E, Harris R, Kamboj MK, Marks S, Nandagopal R, Page L et al An endocrine perspective on menstrual suppression for adolescents: achieving good suppression while optimizing bone health. J Pediatr Endocrinol Metab 2021;34:1355–1369. 34388330 10.1515/jpem-2020-0539

[dead206-B87] Landersoe SK , Birch Petersen K, Sørensen AL, Larsen EC, Martinussen T, Lunding SA, Kroman MS, Nielsen HS, Nyboe Andersen A. Ovarian reserve markers after discontinuing long-term use of combined oral contraceptives. Reprod Biomed Online 2020;40:176–186. 31831368 10.1016/j.rbmo.2019.10.004

[dead206-B88] Laufer MR , Einarsson JI. Surgical management of superficial peritoneal adolescent endometriosis. J Pediatr Adolesc Gynecol 2019;32:339–341. 30708067 10.1016/j.jpag.2019.01.005

[dead206-B89] Li C , Yin X, Liu Z, Wang J. Emerging potential mechanism and therapeutic target of ferroptosis in PDAC: a promising future. Int J Mol Sci 2022;23:15031. 36499358 10.3390/ijms232315031 PMC9740869

[dead206-B90] Li Y , He Y, Cheng W, Zhou Z, Ni Z, Yu C. Double-edged roles of ferroptosis in endometriosis and endometriosis-related infertility. Cell Death Discov 2023;9:306. 37607902 10.1038/s41420-023-01606-8 PMC10444804

[dead206-B91] Lidegaard O , Nielsen LH, Skovlund CW, Løkkegaard E. Venous thrombosis in users of non-oral hormonal contraception: follow-up study, Denmark 2001-10. BMJ 2012;344:e2990. 22577198 10.1136/bmj.e2990 PMC3349780

[dead206-B92] Lidegaard Ø , Nielsen LH, Skovlund CW, Skjeldestad FE, Løkkegaard E. Risk of venous thromboembolism from use of oral contraceptives containing different progestogens and oestrogen doses: Danish cohort study, 2001-9. BMJ 2011;343:d6423. 22027398 10.1136/bmj.d6423 PMC3202015

[dead206-B93] Lindsay PC , Shaw RW, Bennink HJ, Kicovic P. The effect of add-back treatment with tibolone (Livial) on patients treated with the gonadotropin-releasing hormone agonist triptorelin (Decapeptyl). Fertil Steril 1996;65:342–348. 8566259 10.1016/s0015-0282(16)58096-0

[dead206-B94] Lovett JL , Chima MA, Wexler JK, Arslanian KJ, Friedman AB, Yousif CB, Strassmann BI. Oral contraceptives cause evolutionarily novel increases in hormone exposure: a risk factor for breast cancer. Evol Med Public Health 2017;2017:97–108. 28685096 10.1093/emph/eox009 PMC5494186

[dead206-B95] Lund CI , Engdahl B, Rosseland LA, Stubhaug A, Grimnes G, Furberg AS, Steingrímsdóttir ÓA, Nielsen CS. The association between age at menarche and chronic pain outcomes in women: the Tromsø Study, 2007 to 2016. Pain 2022;163:1790–1799. 35239542 10.1097/j.pain.0000000000002579 PMC9393800

[dead206-B96] Ma XQ , Liu YY, Zhong ZQ, Chen SM, Hu WT, Sheng YR, Liu YK, Wei CY, Li MQ, Zhu XY. Heme induced progesterone-resistant profiling and promotion of endometriosis in vitro and in vivo. Biochim Biophys Acta Mol Basis Dis 2023;1869:166761. 37247698 10.1016/j.bbadis.2023.166761

[dead206-B97] MacGregor EA , Guillebaud J. The 7-day contraceptive hormone-free interval should be consigned to history. BMJ Sex Reprod Health 2018;44:214–220. 10.1136/bmjsrh-2017-200036 29945924

[dead206-B98] Malvezzi M , Carioli G, Rodriguez T, Negri E, La Vecchia C. Global trends and predictions in ovarian cancer mortality. Ann Oncol 2016;27:2017–2025. 27597548 10.1093/annonc/mdw306

[dead206-B99] Manzoli L , De Vito C, Marzuillo C, Boccia A, Villari P. Oral contraceptives and venous thromboembolism: a systematic review and meta-analysis. Drug Saf 2012;35:191–205. 22283630 10.2165/11598050-000000000-00000

[dead206-B100] Mandelbaum RS , Melville SJF, Violette CJ, Guner JZ, Doody KA, Matsuzaki S, Quinn MM, Ouzounian JG, Paulson RJ, Matsuo K. The association between uterine adenomyosis and adverse obstetric outcomes: a propensity score-matched analysis. Acta Obstet Gynecol Scand 2023;102:833–842. doi: 10.1111/aogs.14581. 37087741 PMC10333655

[dead206-B101] Mansour D , Westhoff C, Kher U, Korver T. Pooled analysis of two randomized, open-label studies comparing the effects of nomegestrol acetate/17β-estradiol and drospirenone/ethinyl estradiol on bleeding patterns in healthy women. Contraception 2017;95:390–397. 28011288 10.1016/j.contraception.2016.12.001

[dead206-B102] Margaritis K , Margioula-Siarkou G, Margioula-Siarkou C, Petousis S, Galli-Tsinopoulou A. Contraceptive methods in adolescence: a narrative review of guidelines. Eur J Contracept Reprod Health Care 2023;28:51–57. 36637987 10.1080/13625187.2022.2162336

[dead206-B103] Martire FG , Lazzeri L, Conway F, Siciliano T, Pietropolli A, Piccione E, Solima E, Centini G, Zupi E, Exacoustos C. Adolescence and endometriosis: symptoms, ultrasound sign and early diagnosis. Fertil Steril 2020;114:1049–1057. 33036795 10.1016/j.fertnstert.2020.06.012

[dead206-B104] Martire FG , Russo C, Selntigia A, Nocita E, Soreca G, Lazzeri L, Zupi E, Exacoustos C. Early noninvasive diagnosis of endometriosis: dysmenorrhea and specific ultrasound findings are important indicators in young women. Fertil Steril 2023;119:455–464. 36493871 10.1016/j.fertnstert.2022.12.004

[dead206-B106] Mayer TG , Neblett R, Cohen H, Howard KJ, Choi YH, Williams MJ, Perez Y, Gatchel RJ. The development and psychometric validation of the central sensitization inventory. Pain Pract 2012;12:276–285. 21951710 10.1111/j.1533-2500.2011.00493.x PMC3248986

[dead206-B107] Millischer AE , Santulli P, Da Costa S, Bordonne C, Cazaubon E, Marcellin L, Chapron C. Adolescent endometriosis: prevalence increases with age on magnetic resonance imaging scan. Fertil Steril 2023;119:626–633. 36592649 10.1016/j.fertnstert.2022.12.039

[dead206-B108] Modugno F , Ness RB, Allen GO, Schildkraut JM, Davis FG, Goodman MT. Oral contraceptive use, reproductive history, and risk of epithelial ovarian cancer in women with and without endometriosis. Am J Obstet Gynecol 2004;191:733–740. 15467532 10.1016/j.ajog.2004.03.035

[dead206-B109] Mørch LS , Skovlund CW, Hannaford PC, Iversen L, Fielding S, Lidegaard Ø. Contemporary hormonal contraception and the risk of breast cancer. N Engl J Med 2017;377:2228–2239. 29211679 10.1056/NEJMoa1700732

[dead206-B110] Morimont L , Jost M, Gaspard U, Foidart JM, Dogné JM, Douxfils J. Low thrombin generation in users of a contraceptive containing estetrol and drospirenone. J Clin Endocrinol Metab 2022;108:135–143. 36099501 10.1210/clinem/dgac511 PMC9759169

[dead206-B111] Murji A , Biberoğlu K, Leng J, Mueller MD, Römer T, Vignali M, Yarmolinskaya M. Use of dienogest in endometriosis: a narrative literature review and expert commentary. Curr Med Res Opin 2020;36:895–907. 32175777 10.1080/03007995.2020.1744120

[dead206-B112] Muzii L , Di Tucci C, Di Feliciantonio M, Galati G, Verrelli L, Donato VD, Marchetti C, Panici PB. Management of endometriomas. Semin Reprod Med 2017;35:25–30. 27926971 10.1055/s-0036-1597126

[dead206-B113] Myszko O , Al-Husayni N, Talib HJ. Painful periods in the adolescent girl. Pediatr Ann 2020;49:e176–e182. 32275762 10.3928/19382359-20200318-01

[dead206-B114] Nalaboff KM , Pellerito JS, Ben-Levi E. Imaging the endometrium: disease and normal variants. Radiographics 2001;21:1409–1424. 11706213 10.1148/radiographics.21.6.g01nv211409

[dead206-B115] Nash Z , Thwaites A, Davies M. Tailored regimens for combined hormonal contraceptives. BMJ 2020;368:m200. 32014854 10.1136/bmj.m200

[dead206-B116] Neblett R , Hartzell MM, Mayer TG, Cohen H, Gatchel RJ. Establishing clinically relevant severity levels for the central sensitization inventory. Pain Pract 2017;17:166–175. 26989894 10.1111/papr.12440

[dead206-B117] Nelson SM , Ewing BJ, Gromski PS, Briggs SF. Contraceptive-specific antimüllerian hormone values in reproductive-age women: a population study of 42,684 women. Fertil Steril 2023;119:1069–1077. 36801456 10.1016/j.fertnstert.2023.02.019

[dead206-B118] Ng SW , Norwitz SG, Taylor HS, Norwitz ER. Endometriosis: the role of iron overload and ferroptosis. Reprod Sci2020a;27:1383–1390. 32077077 10.1007/s43032-020-00164-z

[dead206-B119] Ng N , Wahl K, Orr NL, Noga H, Williams C, Allaire C, Bedaiwy MA, Yong PJ. Endometriosis and negative perception of the medical profession. J Obstet Gynaecol Can2020b;42:248–255. 31864912 10.1016/j.jogc.2019.08.034

[dead206-B120] Orr NL , Albert A, Liu YD, Lum A, Hong J, Ionescu CL, Senz J, Nazeran TM, Lee AF, Noga H et al KRAS mutations and endometriosis burden of disease. J Pathol Clin Res2023a;9:302–312. 36977195 10.1002/cjp2.317 PMC10240146

[dead206-B121] Orr NL , Huang AJ, Liu YD, Noga H, Bedaiwy MA, Williams C, Allaire C, Yong PJ. Association of central sensitization inventory scores with pain outcomes after endometriosis surgery. JAMA Netw Open2023b;6:e230780. 36848090 10.1001/jamanetworkopen.2023.0780 PMC9972194

[dead206-B122] Orr NL , Wahl KJ, Lisonek M, Joannou A, Noga H, Albert A, Bedaiwy MA, Williams C, Allaire C, Yong PJ. Central sensitization inventory in endometriosis. Pain 2022;163:e234–e245. 34030173 10.1097/j.pain.0000000000002351

[dead206-B123] Orr NL , Wahl KJ, Noga H, Allaire C, Williams C, Bedaiwy MA, Albert A, Smith KB, Yong PJ. Phenotyping sexual pain in endometriosis using the central sensitization inventory. J Sex Med 2020;17:761–770. 31983669 10.1016/j.jsxm.2019.12.019

[dead206-B124] Ott MA , Sucato GS; Committee on Adolescence. Contraception for adolescents. Pediatrics 2014;134:e1257–e1281. 25266435 10.1542/peds.2014-2300

[dead206-B125] Parazzini F , Roncella E, Cipriani S, Trojano G, Barbera V, Herranz B, Colli E. The frequency of endometriosis in the general and selected populations: a systematic review. J Endometriosis Pelvic Pain Disord 2020;12:176–189.

[dead206-B126] Park BY , Yao R, Rossi J, Lee AW. Severe maternal morbidity associated with endometriosis: a population based retrospective cohort study. Fertil Steril 2023;120:360–368. doi:10.1016/j.fertnstert.2023.03.033. 37030633

[dead206-B127] Pino I , Belloni GM, Barbera V, Solima E, Radice D, Angioni S, Arena S, Bergamini V, Candiani M, Maiorana A et al; “Endometriosis Treatment Italian Club” (ETIC). “Better late than never but never late is better”, especially in young women. A multicenter Italian study on diagnostic delay for symptomatic endometriosis. Eur J Contracept Reprod Health Care 2023;28:10–16. 36287190 10.1080/13625187.2022.2128644

[dead206-B128] Powell A. Choosing the right oral contraceptive pill for teens. Pediatr Clin North Am 2017;64:343–358. 28292450 10.1016/j.pcl.2016.11.005

[dead206-B129] Practice Committee of American Society for Reproductive Medicine. Treatment of pelvic pain associated with endometriosis. Fertil Steril 2008;90:S260–S269. 19007642 10.1016/j.fertnstert.2008.08.057

[dead206-B130] Praetorius TH , Leonova A, Lac V, Senz J, Tessier-Cloutier B, Nazeran TM, Köbel M, Grube M, Kraemer B, Yong PJ et al Molecular analysis suggests oligoclonality and metastasis of endometriosis lesions across anatomically defined subtypes. Fertil Steril 2022;118:524–534. 35715244 10.1016/j.fertnstert.2022.05.030

[dead206-B131] Raimondo D , Raffone A, Renzulli F, Sanna G, Raspollini A, Bertoldo L, Maletta M, Lenzi J, Rovero G, Travaglino A et al Prevalence and risk factors of central sensitization in women with endometriosis. J Minim Invasive Gynecol 2023;30:73–80.e1. 36441085 10.1016/j.jmig.2022.10.007

[dead206-B132] Rasp E , Saavalainen L, But A, Gissler M, Härkki P, Heikinheimo O, Rönö K. Surgically confirmed endometriosis in adolescents in Finland-A register-based cross-sectional cohort study. Acta Obstet Gynecol Scand 2022;101:1065–1073. 35818936 10.1111/aogs.14419 PMC9812065

[dead206-B133] Renfree MB. Why menstruate? Bioessays 2012;34:1. 22170468 10.1002/bies.201190071

[dead206-B134] Roden RC. Reversible interventions for menstrual management in adolescents and young adults with gender incongruence. Ther Adv Reprod Health 2023;17:26334941231158251. 36938373 10.1177/26334941231158251 PMC10017940

[dead206-B135] Sarıdoğan E. Adolescent endometriosis. Eur J Obstet Gynecol Reprod Biol 2017;209:46–49. 27342684 10.1016/j.ejogrb.2016.05.019

[dead206-B136] Saridoğan E. Endometriosis in teenagers. Womens Health (Lond Engl) 2015;11:705–709. 26315257 10.2217/whe.15.58

[dead206-B137] Scerbo T , Colasurdo J, Dunn S, Unger J, Nijs J, Cook C. Measurement properties of the central sensitization inventory: a systematic review. Pain Pract 2018;18:544–554. 28851012 10.1111/papr.12636

[dead206-B138] Shah DK , Missmer SA. Scientific investigation of endometriosis among adolescents. J Pediatr Adolesc Gynecol 2011;24:S18–9. 21856546 10.1016/j.jpag.2011.07.008

[dead206-B139] Short RV. The evolution of human reproduction. Proc R Soc Lond B Biol Sci 1976;195:3–24. 13383 10.1098/rspb.1976.0095

[dead206-B140] Stegeman BH , de Bastos M, Rosendaal FR, van Hylckama Vlieg A, Helmerhorst FM, Stijnen T, Dekkers OM. Different combined oral contraceptives and the risk of venous thrombosis: systematic review and network meta-analysis. BMJ 2013;347:f5298. 24030561 10.1136/bmj.f5298 PMC3771677

[dead206-B141] Straker LM , Hall GL, Mountain J, Howie EK, White E, McArdle N, Eastwood PR; Raine Study 22 Year Follow-Up Investigator Group. Rationale, design and methods for the 22 year follow-up of the Western Australian Pregnancy Cohort (Raine) Study. BMC Public Health 2015;15:663. 26169918 10.1186/s12889-015-1944-6 PMC4501054

[dead206-B142] Straub RH. The complex role of estrogens in inflammation. Endocr Rev 2007;28:521–574. 17640948 10.1210/er.2007-0001

[dead206-B143] Suda K , Nakaoka H, Yoshihara K, Ishiguro T, Tamura R, Mori Y, Yamawaki K, Adachi S, Takahashi T, Kase H et al Clonal expansion and diversification of cancer-associated mutations in endometriosis and normal endometrium. Cell Rep 2018;24:1777–1789. 30110635 10.1016/j.celrep.2018.07.037

[dead206-B144] Sulak PJ , Kuehl TJ, Coffee A, Willis S. Prospective analysis of occurrence and management of breakthrough bleeding during an extended oral contraceptive regimen. Am J Obstet Gynecol 2006;195:935–941. 16647684 10.1016/j.ajog.2006.02.048

[dead206-B145] Tandoi I , Somigliana E, Riparini J, Ronzoni S, Vigano P, Candiani M. High rate of endometriosis recurrence in young women. J Pediatr Adolesc Gynecol 2011;24:376–379. 21906976 10.1016/j.jpag.2011.06.012

[dead206-B146] Taskin O , Yalcinoglu AI, Kucuk S, Uryan I, Buhur A, Burak F. Effectiveness of tibolone on hypoestrogenic symptoms induced by goserelin treatment in patients with endometriosis. Fertil Steril 1997;67:40–45. 8986681 10.1016/s0015-0282(97)81853-5

[dead206-B147] Taylor HS , Adamson GD, Diamond MP, Goldstein SR, Horne AW, Missmer SA, Snabes MC, Surrey E, Taylor RN. An evidence-based approach to assessing surgical versus clinical diagnosis of symptomatic endometriosis. Int J Gynaecol Obstet 2018;142:131–142. 29729099 10.1002/ijgo.12521

[dead206-B148] Tepper NK , Dragoman MV, Gaffield ME, Curtis KM. Nonoral combined hormonal contraceptives and thromboembolism: a systematic review. Contraception 2017;95:130–139. 27771476 10.1016/j.contraception.2016.10.005 PMC11025291

[dead206-B149] Thomas SL , Ellertson C. Nuisance or natural and healthy: should monthly menstruation be optional for women? Lancet 2000;355:922–924. 10752720 10.1016/S0140-6736(99)11159-0

[dead206-B151] Treloar SA , Bell TA, Nagle CM, Purdie DM, Green AC. Early menstrual characteristics associated with subsequent diagnosis of endometriosis. Am J Obstet Gynecol 2010;202:534.e1–534.e6. 10.1016/j.ajog.2009.10.857 20022587

[dead206-B150] Tucker DR , Noga HL, Lee C, Chiu DS, Bedaiwy MA, Williams C, Allaire C, Talhouk A, Yong PJ. Pelvic pain comorbidities associated with quality of life after endometriosis surgery. Am J Obstet Gynecol 2023;229:147.e1–147.e20. 10.1016/j.ajog.2023.04.040 37148956

[dead206-B152] Ulukus M , Cakmak H, Arici A. The role of endometrium in endometriosis. J Soc Gynecol Investig 2006;13:467–476. 10.1016/j.jsgi.2006.07.005 16990031

[dead206-B153] Unger CA , Laufer MR. Progression of endometriosis in non-medically managed adolescents: a case series. J Pediatr Adolesc Gynecol 2011;24:e21–3–e23. 21126894 10.1016/j.jpag.2010.08.002

[dead206-B154] Vannuccini S , Luisi S, Tosti C, Sorbi F, Petraglia F. Role of medical therapy in the management of uterine adenomyosis. Fertil Steril 2018;109:398–405. 29566852 10.1016/j.fertnstert.2018.01.013

[dead206-B155] Vercellini P , Bandini V, Buggio L, Barbara G, Berlanda N, Dridi D, Frattaruolo MP, Somigliana E. Mitigating the economic burden of GnRH agonist therapy for progestogen-resistant endometriosis: why not? Hum Reprod Open 2023a;2023:hoad008. doi:10.1093/hropen/hoad008. PMC10067151 37016694

[dead206-B1600] Vercellini P, Bandini V, Vigano P, Di Stefano G, Merli CEM, Somigliana E. Proposal for targeted, neo-evolutionary-oriented, secondary prevention of early-onset endometriosis and adenomyosis. Part 1: Pathogenic aspects. *Hum Reprod* 2024;39 1. 10.1093/humrep/dead229 PMC10876119 37951243

[dead206-B156] Vercellini P , Barbara G, Somigliana E, Bianchi S, Abbiati A, Fedele L. Comparison of contraceptive ring and patch for the treatment of symptomatic endometriosis. Fertil Steril 2010;93:2150–2161. 19328469 10.1016/j.fertnstert.2009.01.071

[dead206-B158] Vercellini P , Buggio L, Berlanda N, Barbara G, Somigliana E, Bosari S. Estrogen-progestins and progestins for the management of endometriosis. Fertil Steril2016a;106:1552–1571.e2. 27817837 10.1016/j.fertnstert.2016.10.022

[dead206-B159] Vercellini P , Buggio L, Frattaruolo MP, Borghi A, Dridi D, Somigliana E. Medical treatment of endometriosis-related pain. Best Pract Res Clin Obstet Gynaecol 2018;51:68–91. 29530425 10.1016/j.bpobgyn.2018.01.015

[dead206-B157] Vercellini P , Donati A, Ottolini F, Frassineti A, Fiorini J, Nebuloni V, Frattaruolo MP, Roberto A, Mosconi P, Somigliana E. Norethindrone acetate or dienogest for the treatment of symptomatic endometriosis: a before and after study. Fertil Steril2016b;109:1086–1043.e3. 10.1016/j.fertnstert.2015.11.016 26677792

[dead206-B160] Vercellini P , Eskenazi B, Consonni D, Somigliana E, Parazzini F, Abbiati A, Fedele L. Oral contraceptives and risk of endometriosis: a systematic review and meta-analysis. Hum Reprod Update 2011;17:159–170. 20833638 10.1093/humupd/dmq042

[dead206-B161] Vercellini P , Viganò P, Bandini V, Buggio L, Berlanda N, Somigliana E. Association of endometriosis and adenomyosis with pregnancy and infertility. Fertil Steril2023c;119:727–740. 36948440 10.1016/j.fertnstert.2023.03.018

[dead206-B162] Vercellini P , Viganò P, Somigliana E, Fedele L. Endometriosis: pathogenesis and treatment. Nat Rev Endocrinol 2014;10:261–275. 24366116 10.1038/nrendo.2013.255

[dead206-B163] Viganò P , Casalechi M, Vercellini P, Somigliana E. “Shadow of a Doubt”-the pathogenic role of endometrial defects in endometriosis development and endometriosis-associated infertility: robust demonstration of clinical relevance is still urgently needed. Biomolecules 2023;13:651. 37189399 10.3390/biom13040651 PMC10135529

[dead206-B164] Vinatier D , Cosson M, Dufour P. Is endometriosis an endometrial disease? Eur J Obstet Gynecol Reprod Biol 2000;91:113–125. 10869783 10.1016/s0301-2115(99)00263-8

[dead206-B165] Wiegers HMG , Scheres LJJ, Tahir L, Hutten BA, Middeldorp S, Mijatovic V. Risk of venous thromboembolism in women with endometriosis. Thromb Res 2022;217:104–106. 35932497 10.1016/j.thromres.2022.07.014

[dead206-B166] Wyatt J , Fernando SM, Powell SG, Hill CJ, Arshad I, Probert C, Ahmed S, Hapangama DK. The role of iron in the pathogenesis of endometriosis: a systematic review. Hum Reprod Open 2023;2023:hoad033. 10.1093/hropen/hoad033 PMC10457727 37638130

[dead206-B167] Wüest A , Limacher JM, Dingeldein I, Siegenthaler F, Vaineau C, Wilhelm I, Mueller MD, Imboden S. Pain levels of women diagnosed with endometriosis: is there a difference in younger women? J Pediatr Adolesc Gynecol 2023;36:140–147. 36343859 10.1016/j.jpag.2022.10.011

[dead206-B168] Xin L , Ma Y, Ye M, Chen L, Liu F, Hou Q. Efficacy and safety of oral gonadotropin-releasing hormone antagonists in moderate-to-severe endometriosis-associated pain: a systematic review and network meta-analysis. Arch Gynecol Obstet 2023;308:1047–1056. 36656435 10.1007/s00404-022-06862-0 PMC10435625

[dead206-B169] Yan H , Shi J, Li X, Dai Y, Wu Y, Zhang J, Gu Z, Zhang C, Leng J. Oral gonadotropin-releasing hormone antagonists for treating endometriosis-associated pain: a systematic review and network meta-analysis. Fertil Steril 2022;118:1102–1116. 36283862 10.1016/j.fertnstert.2022.08.856

[dead206-B170] Yılmaz Hanege B , Güler Çekıç S, Ata B. Endometrioma and ovarian reserve: effects of endometriomata per se and its surgical treatment on the ovarian reserve. Facts Views Vis Obgyn 2019;11:151–157. 31824636 PMC6897522

[dead206-B171] Yong PJ , Alsowayan N, Noga H, Williams C, Allaire C, Lisonkova S, Bedaiwy MA. CHC for pelvic pain in women with endometriosis: ineffectiveness or discontinuation due to side-effects. Hum Reprod Open 2020;2020:hoz040. 10.1093/hropen/hoz040 PMC7048681 32128454

[dead206-B172] Youngster M , Laufer MR, Divasta AD. Endometriosis for the primary care physician. Curr Opin Pediatr 2013;25:454–462. 23817302 10.1097/MOP.0b013e3283628092

[dead206-B173] Younis JS , Shapso N, Fleming R, Ben-Shlomo I, Izhaki I. Impact of unilateral versus bilateral ovarian endometriotic cystectomy on ovarian reserve: a systematic review and meta-analysis. Hum Reprod Update 2019;25:375–391. 30715359 10.1093/humupd/dmy049

[dead206-B174] Zakhari A , Delpero E, McKeown S, Tomlinson G, Bougie O, Murji A. Endometriosis recurrence following post-operative hormonal suppression: a systematic review and meta-analysis. Hum Reprod Update 2021;27:96–107. 33020832 10.1093/humupd/dmaa033 PMC7781224

[dead206-B175] Zannoni L , Giorgi M, Spagnolo E, Montanari G, Villa G, Seracchioli R. Dysmenorrhea, absenteeism from school, and symptoms suspicious for endometriosis in adolescents. J Pediatr Adolesc Gynecol 2014;27:258–265. 24746919 10.1016/j.jpag.2013.11.008

[dead206-B176] Zigler RE , McNicholas C. Unscheduled vaginal bleeding with progestin-only contraceptive use. Am J Obstet Gynecol 2017;216:443–450. 27988268 10.1016/j.ajog.2016.12.008

[dead206-B177] Zorbas KA , Economopoulos KP, Vlahos NF. Continuous versus cyclic oral contraceptives for the treatment of endometriosis: a systematic review. Arch Gynecol Obstet 2015;292:37–43. 25644508 10.1007/s00404-015-3641-1

